# Aging-related lateral ventricular shape changes and corresponding mechanical loading derived from longitudinal image registration

**DOI:** 10.1007/s10237-026-02080-8

**Published:** 2026-06-03

**Authors:** Lauren Cunniff, Johannes Weickenmeier, the Alzheimer’s Disease Neuroimaging Initiative

**Affiliations:** 1 Department of Mechanical Engineering, Stevens Institute of Technology, Hoboken, NJ 07030 USA; 2 Department of Engineering Science, University of Oxford, Oxford, OX3 7DQ UK; 3 Podium Institute for Sports Medicine and Technology, University of Oxford, Oxford, OX3 7DQ UK

**Keywords:** Longitudinal nonlinear image registration, Ventricular enlargement, Ventricular shape change, Ventricular wall loading, White matter hyperintensities

## Abstract

Lateral ventricular enlargement is one of the most prominent features of the aging brain and is clearly visible on structural magnetic resonance imaging. Both longitudinal and cross-sectional imaging studies have shown that ventricular volume progressively increases with age and expands even faster in neurodegenerative diseases such as Alzheimer’s disease and related dementias. Strikingly, however, we only have a limited understanding of ventricular shape changes and the corresponding mechanical loads that act on the ventricular wall as we age. Therefore, we propose a framework that uses nonlinear registration to quantify subject-specific brain deformations between two longitudinal scans, maps the resulting warp field onto a ventricular surface template mesh, and quantifies mechanical loading measures including displacement magnitude, curvature change, area stretch, and maximum principal wall strain. From the Alzheimer’s Disease Neuroimaging Initiative, we selected a cohort of 50 cognitively normal subjects aged 70–75 years at baseline and with a follow-up scan 4–5 years later. In this group, we observed mostly uniform expansion of the lateral ventricles with an average displacement magnitude of 0.88 ± 0.3 mm across the whole ventricle. At the same time, there are distinct sections of the ventricular wall that experience high mechanical loads with respect to our mechanomarkers. Specifically, maximum mechanical loading consistently localizes along the ventricular edges and atrium while the ventricle’s main body exhibits minimal loading. Based on the cohort included in this study, we did not observe sex-based differences with respect to any mechanomarker, noticed that on average 29.2 ± 9.3% of the ventricular wall experience wall area increase, and that on average only 4.4 ± 2.5% of the ventricular wall experience wall shrinking. Interestingly, regions of elevated mechanical loading showed reliable spatial correspondence with periventricular white matter hyperintensity locations in our subjects for whom FLAIR imaging was available (n = 39). Additionally, mechanomarkers showed increased magnitudes with periventricular white matter hyperintensity burden, with curvature change demonstrating the strongest group separation. These findings suggest that ventricular enlargement is associated with localized mechanical stresses that coincide with vulnerable white matter regions. Taken together, we present strong evidence in support of the hypothesis that the mechanical loading associated with age-related ventricular enlargement is intricately linked to periventricular white matter degeneration and corresponding cognitive decline.

**Supplementary Information:**

The online version contains supplementary material available at 10.1007/s10237-026-02080-8.

## Introduction

Lateral ventricular enlargement is one of the most prominent brain shape change features associated with aging. The underlying grey and white matter degeneration is tightly linked to gradual cognitive and functional decline in the elderly (Todd et al. 2018; Resnick et al. 2003). In addition, many neurodegenerative diseases accelerate neuropathology throughout the brain and, therefore, exacerbate ventricular expansion when compared with healthy aging (Carmichael et al. 2007; Tang et al. 2014). Conveniently, ventricular changes are significantly more pronounced and easier to detect in comparison to most other features of cerebral atrophy including cortical thinning, sulcal widening, and tissue volume loss and can, therefore, be more readily detected in cross-sectional and longitudinal imaging (Blinkouskaya et al. 2021). For example, ventricular volume trajectories of healthy subjects compared to Alzheimer’s disease subjects diverge as early as 40 years of age (Coupé et al. 2019). This suggests that early white and grey matter changes–that are likely undetectable for themselves–aggregate to have a measurable impact on ventricular volume and shape. Additionally, ventricular shapes vary between subjects which could explain the range of pathological presentations observed in magnetic resonance images (MRI). To date, however, only a few methods exist to quantify longitudinal, image-based morphometry-derived shape changes of subcortical structures, such as the lateral ventricles, and no work has been presented to quantify the resulting mechanical loads on the ventricular surface across multiple subjects. Clinically, such a method would represent a useful diagnostic tool to detect abnormal brain shape changes caused by various neurodegenerative diseases and distinguish accelerated changes from healthy aging.

Anatomically, the ventricular epithelium is lined by the ependymal wall that is composed of four distinct layers with varying thicknesses and densities. Going from ventricle towards brain parenchyma, one observes a monolayer of cuboidal multiciliated ependymal cells (Layer I), a prominent hypocellular gap rich in processes from ependymal cells and astrocytes (Layer II), a ribbon of cells composed of astrocytes (Layer III), and a transitional zone into the brain parenchyma (Layer IV) (Quiñones-Hinojosa et al. 2006; Del Bigio 2010). The multiciliated ependymal cells in Layer I are tightly joined by connexins and cadherins, or gap junction proteins, which form tight inter-cellular connections (Oliver et al. 2013). Functionally, the ventricular wall is a bidirectional barrier and transport system for cerebrospinal fluid and interstitial fluid exchange (Del Bigio 2010; Roales-Buján et al. 2012) with the goal to remove waste products from the parenchyma and create a homeostatic environment favorable for the function and protection of periventricular white matter (Johanson et al. 2011; Jiménez et al. 2014)). Layer II is present in the entire ependymal wall in the lateral ventricles but varies in thickness from region to region. It has also been observed that aging can cause degeneration on layers I and II in the form of denudation of the ependymal layer (Shook et al. 2014; Todd et al. 2018; Visser et al. 2021). In respective sections of the ventricular wall, loss of the ependymal barrier is accompanied by astrogliosis in layer III and the formation of a scarred barrier that has substantially lower functional ability to regulate fluid and substance exchange at the cerebrospinal fluid-brain interface (Shook et al. 2014; Todd et al. 2018).

Traditional morphometry based on structural MRI primarily quantifies geometric and volumetric properties of brain structures, such as regional tissue volumes and shape features (Ashburner et al. 1998; Ashburner and Friston 2000). Techniques such as voxel-based morphometry (VBM) allow voxel-wise comparison of local grey and white matter volumes between groups or across the lifespan (Ashburner and Friston 2000; Sotiras et al. 2013). These approaches are designed to detect structural differences and characterize patterns of atrophy or enlargement. However, they mostly just describe how much a structure changes, rather than how such changes mechanically affect surrounding tissue. In contrast, mechanics-based markers aim to interpret anatomical change in terms of tissue deformation and loading, providing a framework to quantify stresses and strains resulting from shape changes. This perspective is particularly relevant in brain aging, neurodegeneration, and injury, where processes such as ventricular enlargement and cerebral atrophy alter the mechanical loading state of the brain and modify deformation patterns in surrounding tissue (Blinkouskaya 2022; Schäfer et al. 2019; Tueni et al. 2026; Pederzoli et al. 2025). Resulting deformation can impose strain on periventricular regions, with elevated ventricular wall strain spatially co-localizing with areas prone to white matter hyperintensities (Caçoilo et al. 2023; Visser et al. 2021). These findings suggest that mechanically induced strain may contribute to structural vulnerability and functional decline. Ventricular volume change has been extensively reported in literature (Apostolova et al. 2012; Trimarchi et al. 2013; Leung et al. 2013; Tang et al. 2014; Coupé et al. 2019). A majority of studies report absolute volumes or volume change obtained from voxel-based morphometry using either cross-sectional (Apostolova et al. 2012; Fujita et al. 2023; Trimarchi et al. 2013) or longitudinal imaging data (Leung et al. 2013; Tang et al. 2014). Registering multiple longitudinal scans can generate simulated sequences demonstrating morphological evolution (Khanal et al. 2017) and provide a powerful method for quantifying shape changes (Fox et al. 2000). It is important to note, however, that most longitudinal data is limited to 1–2 year observation periods, which provide only short-term changes rather than a useful perspective on aging. Other scalar measures related to ventricular dimensions have been proposed, including the Evans index or Frontal Horn Ratio, which relate the maximum width of the frontal horns to the maximal internal diameter of the skull of the brain, respectively (Kwon et al. 2014; Brix et al. 2017). Beyond volumetric measures, statistical shape-based approaches–including tensor-based morphometry, radial distance metrics, and surface-based analyses–have been used to characterize ventricular and hippocampal deformation with high sensitivity (Wang et al. 2011; Ferrarini et al. 2006; Chou et al. 2008; Shi et al. 2015). These methods enable precise tracking of morphological change and differentiation between normal and pathological aging (Gutman et al. 2023; Apostolova et al. 2012; Fjell et al. 2009). For instance, Gutman et al. used Linear Discriminant Analysis (LDA) to enhance the sensitivity of ventricular shape features for clinical trials (Gutman et al. 2023). Similarly, Wang et al. utilized tensor-based morphometry (mTBM) and radial distance metrics to quantify hippocampal and ventricular atrophy (Wang et al. 2011). However, both volumetric and shape-based approaches remain purely descriptive, focusing on structural change without quantifying the impact of ventricular expansion on periventricular tissue stress and strain. There are only few examples that aim at characterizing actual shape change features using variations of surface distance-based approaches (Ferrarini et al. 2006; Chou et al. 2008; Shi et al. 2015). These methods typically create a reference mesh of the ventricular surface first, then use node-wise mesh perturbation to find a match on either a subject-specific or group-averaged realization of the ventricular surface. They then typically report on either differences between subject groups, e.g., differences between cognitively normal, mild cognitive impairment, and Alzheimer’s disease subjects (Ferrarini et al. 2006; Apostolova et al. 2012) or shape change features within subjects between scans (Fjell et al. 2009). These approaches all provide discrete, non-smooth maps related to shape change but do not quantify the degree of cellular loading arising from ventricular expansion. In contrast, nonrigid image registration methods–which we used here–provide spatially resolved smooth measures of tissue deformation. In conventional registration, the resulting warp field is a kinematic mapping that aligns images by optimizing similarity and regularization criteria (Sotiras et al. 2013; Ashburner 2007), without explicitly enforcing physical conservation laws or material behavior (Sotiras et al. 2013; Reithmeir et al. 2026). While mechanics-driven registration frameworks offer a more physically consistent alternative (Amiri-Hezaveh et al. 2025), they are not yet widely adopted in longitudinal neuroimaging due to high computational cost.

The considerable variability of ventricular volume change across the healthy and diseased population indicates that volume alone is an unreliable diagnostic marker for Alzheimer’s disease (Nestor et al. 2008). Moreover, while many statistical shape modeling techniques allow for high precision tracking of ventricular deformation, these methods do not provide insights into the resulting local tissue loading (Gutman et al. 2023; Wang et al. 2011). In the present work, we address these gaps and move beyond traditional volumetric and statistical shape analyses by introducing a biomechanical framework for tracking ventricular loading over time. More specifically, our present work introduces a framework to quantify mechanomarkers of ventricular expansion based on nonlinear image registration of longitudinal image data. We enable direct comparison between subjects by interpolating a template 3D ventricular surface model in each subject’s registration output and determine several mechanical properties associated with shape change. From the Alzheimer’s Disease Neuroimaging Initiative dataset, we selected a cohort of 50 subjects that have a baseline scan at around 70 years old and a follow-up scan 4–5 years later. We determine ventricular deformations via nonlinear registration and subsequently compute our mechanomarkers which include ventricular surface curvature change, area stretch, and maximum principal wall strain to assess these measures’ spatial distribution across the ventricle. Lastly, we identify ventricular wall sections that experience peak mechanical loading and demonstrate that these locations spatially overlap with periventricular white matter hyperintensity regions.

## Methods

### Subject selection

We obtained all imaging data from the Alzheimer’s Disease Neuroimaging Initiative (ADNI) database (adni.loni.usc.edu) (Petersen et al. 2010; Jack et al. 2008). Among all 892 cognitively normal subjects across all ADNI studies, i.e., ADNI1, ADNI-GO, ADNI2, and ADNI3, we applied the following selection criteria: (i) subjects had to be between 70.0 and 75.0 years old at their baseline scan, (ii) have a follow-up scan 4–5 years later, and (iii) must be diagnosed as cognitively healthy at both visits. We ranked the resulting 91 subjects (42 female and 49 male) based on years between scans and selected the 50 subjects (25 female and 25 male) with the longest spacing. We excluded the remaining 41 subjects to have equal number of male and female subjects for statistical analysis while maximizing the time gap between scans in our cohort. A subset of 39 subjects (18 female and 21 male) from our cohort had T2 FLAIR images available at both baseline and follow-up and were included in our white matter hyperintensity analysis. Each subject’s baseline and follow-up scan were visually inspected for artifacts and abnormal features. Table  1 summarizes demographic data of our subjects including age, education, Mini Mental State Examination (MMSE) score, ethnicity, and race. With respect to comorbidities, 6 subjects reported a cardiovascular condition, 16 reported to smoke (although 18 did not specify), and 3 subjects reported a neurologic condition other than a cognitive disorder.

**Table 1:** Summary of our cohort’s demographic data at baseline and follow-up. MMSE refers to the Mini-Mental State Examination score

	All subjects	Female subjects	Male subjects
	(n = 50)	(n = 25)	(n = 25)
Age at baseline [years]	72.0 ± 1.4	72.0 ± 1.6	71.9 ± 1.3
Age at follow-up [years]	76.2 ± 1.4	76.3 ± 1.5	76.1 ± 1.3
Education [years]	16.6 ± 2.2	16.3 ± 2.1	16.8 ± 2.3
MMSE at baseline	29.3 ± 1.0	29.5 ± 0.7	29.3 ± 1.1
MMSE at follow-up	29.0 ± 1.2	28.9 ± 1.4	28.5 ± 1.4
Ethnicity [n]			
Hispanic/Latino	2 (4%)	1 (4%)	1 (4%)
Non-Hispanic/Non-Latino	48 (96%)	24 (96%)	24 (96%)
Race [n]			
White	45 (90%)	22 (88%)	23 (92%)
Black/African American	4 (8%)	2 (8%)	2 (8%)
Multiracial	1 (2%)	1 (4%)	-

### Preprocessing of our longitudinal image dataset

For the registration step to produce accurate brain shape changes between baseline and follow-up, we applied a series of preprocessing steps to all images. First, we account for changes related to image acquisition and reconstruction ranging from possible hardware changes, scanning software versions, and imaging parameters which determine slice thickness, field of view, and matrix size and can cause difference in image resolution, contrast, and detail. Afterwards, we adjust each subject’s two images to remove rigid body motion between baseline and follow-up. Our registration framework is fully based on algorithms available through the FMRIB Software Library (Jenkinson et al. 2012). We first apply *robustfov* to remove the neck and lower head from each image. This step unifies the field of view across our image dataset by discarding unnecessary non-brain regions and improves the quality of subsequent preprocessing steps including brain extraction (Smith 2002). We then apply the automated segmentation tool (*FAST*) to correct for spatial intensity variations which can cause same tissue types to have varying grey scale distributions (Zhang et al. 2001). By removing the low-frequency nonuniform intensity bias field, the quality of intensity-based segmentation steps, such as skull stripping, are substantially improved (Juntu et al. 2005). The images in our dataset had varying dimensions and voxel resolutions, particularly following the preprocessing step using *robustfov*. To ensure consistent image size and resolution across subjects and time points, all images were resampled using the linear image registration tool, *FLIRT*, (Jenkinson and Smith 2001). A reference image size of 211 $\times$ 240 $\times$ 170 voxels at 1 mm isotropic resolution was selected, corresponding to the most frequently encountered image dimension within our cohort. All images were rigidly aligned to a reference image of this size and resolution. Images were stored in NIfTI format and follow the standard RAS (Right–Anterior–Superior) anatomical coordinate convention. In a final preprocessing step, we stripped the skull from each image using the brain extraction tool, *BET*. *BET* has been shown to produce significantly fewer errors relative to other methods when working with T1-weighted MRI scans and produce more smoothed volumes compared to manual segmentation (Fennema-Notestine et al. 2006).

### Reference model of the lateral ventricle

**Fig. 1:**
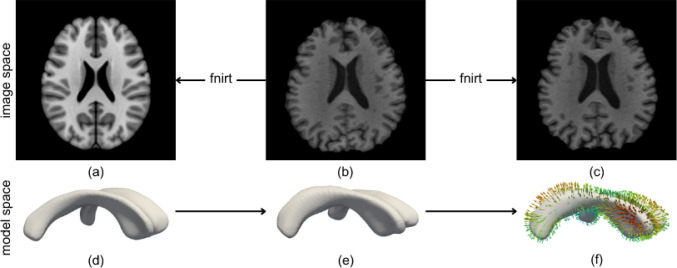
The registration process allows us to map a subject’s ventricular deformations between baseline **b** and follow-up **c** to a common template model (**a**/**d**) for quantitative comparison of ventricular shape changes within our cohort. To that end, we first deform a ventricular surface template model to match each subject’s baseline geometry (**e**). Then, the baseline scan is registered with the follow-up scan to obtain shape changes between scans. These registration results are mapped onto the template mesh to analyze the mechanical loading of the ventricular wall (**f**)

To extract each subject’s lateral ventricular deformation as well as to compare deformations among our subjects, we created a reference model based on the MNI-152 brain template which was created from 3D brain MRI images of 152 cognitively normal subjects (66 females, 86 males) aged between 18 and 44 years (Fonov et al. 2011). We imported this standard-space T1-weighted average structural template into the ScanIP module (version U−2022.12-SP2) of the commercial Simpleware software program (Synposis, Mountain View, CA). Following preliminary intensity-based segmentation of the lateral ventricle, we manually adjusted the segmentation to only include the anterior horns, main body, and atrium, as shown in Fig. 1d; for further clarification about ventricular subregions referenced throughout the present work, we refer to Fig. S1 in the supplementary materials. We use these subdivisions to describe where mechanical changes localize across the ventricular wall and primarily differentiate between the (superior and inferior sections of the) main body, the left and right edges of the main body, the atrium, and the anterior horns. Manual adjustment consisted of local correction in the anterior horns as well as the inferior atrium, followed by Gaussian smoothing of the entire mask with an isotropic kernel of 2 mm (equivalent to 2 voxels in every direction). The resulting ventricle mask contained 21,622 voxels, i.e., 21.622 cm$^3$. From the voxelized mask, ScanIP generated a smooth triangular surface representation, as shown in Fig. 1d. We set both minimum and maximum element edge to be 1 mm to match the resolution of our images to create a highly regular mesh with a uniform distribution of vertices across the ventricular surface. The resulting mesh consists of 16,492 vertices and 32,980 elements.

### Image registration

Figure 1 shows our proposed approach which includes the two separate registration steps that we applied to the data of each subject. We used FMRIB’s linear and nonlinear image registration tools, i.e., *FLIRT* and *FNIRT*, respectively, to align 1) the template image with each subject’s baseline scan and 2) each subject’s baseline and follow-up image. Whenever we apply *FLIRT*, we use skull-stripped images to maximize the alignment between the brain itself; whenever we apply *FNIRT*, we use full-brain images, however, as the skull provides a rigid boundary that improves registration results especially around the cortical surface.

*Template to Baseline*      The MNI152 brain template was registered to each subject’s baseline image such that we could deform the ventricular surface model to align with each subject’s ventricle at baseline, see Fig. 1. We first apply affine transformation with *FLIRT* although we register the baseline to the template as that transformation provides substantially better results that the other direction. We then invert the mapping and use it as the initial guess for the nonlinear registration step. Specifically, we apply *FNIRT* to register the template image (fixed image) to the subjects’ baseline scans (moving image) as FNIRT estimates a warp field that maps the fixed image to the moving image. We then interpolate the template mesh in the registration-derived warp field to obtain a deformed ventricular surface mesh that closely approximates each subject’s ventricle at baseline.

*Baseline to follow-up*      First, we use *FLIRT* to rigidly align the follow-up with the baseline scan in order to remove any misalignment from positional differences between baseline and follow-up scans that are 4–5 years apart. We then obtain the affine transformation map between baseline to the rigidly-aligned follow-up scan using FLIRT which we use as the initial guess for the final nonlinear registration step. At the end, *FNIRT* provides the warp field that maps the baseline scan onto the follow-up scan and captures how the brain deforms between both scans. To quantify ventricular deformation, we interpolate the subject-specific ventricular surface meshes in the respective *FNIRT* output fields and store the ventricular deformation field for each subject, see Fig.1.

### Mechanical evaluation of ventricular deformation

We use the registration-derived subject-specific ventricular displacement field to determine the degree of mechanical loading on the ventricular wall. Specifically, we compute volume and surface area change, surface area stretch, curvature change, and maximum principal wall strain, as approximate measures for the mechanical loading of the ventricular wall’s ependymal cell layer.

*Ventricular volume and surface area*      To compute the volume, we determine the undeformed volume mesh at baseline and the deformed volume mesh at follow-up. We calculate the volume of each tetrahedron element based on the scalar triple product given by (Vince 2005)

1 $$\begin{aligned} V_{\text {tet}} = \frac{1}{6} \left| \overrightarrow{AD} \cdot (\overrightarrow{AB} \times \overrightarrow{AC})\right| , \end{aligned}$$

with edge vectors $\overrightarrow{AB},\,\overrightarrow{AC},\, \text {and}\,\overrightarrow{AD}$. We determine the ventricle’s total surface area by summing over all elements. For each element, we calculate the cross product between the two edge vectors spanning the area which is given by (De Berg et al. 2008)

2 $$\begin{aligned} A_{\text {tri}} = \frac{1}{2} \Vert (\overrightarrow{AB} \times \overrightarrow{AC}) \Vert . \end{aligned}$$

To evaluate total area change and local area stretch, we compute the ventricular surface area for both the undeformed and deformed configuration.

*Ventricular surface curvature change*      Curvature change is a marker for the degree of shape change and, therefore, indicative of the loading that respective regions experience. We define curvature change as a node-wise measure of the difference between surface curvature at baseline and follow-up.

We compute the curvature at each node of the surface mesh from local principal curvatures. To that end, we compute each vertex’s surface normal by averaging the normal vector of all connected elements weighted by their surface area. Since the mesh is a smoothed continuous surface mesh, the surface can be approximated by the biquadratic surface patch in Eq. 3 where *u* and *v* refer to the local coordinate system determined by the normal and tangent vectors at the vertex (Sacks et al. 1999). We employ a least-squares approach to fit the patch for a more precise calculation.

3 $$\begin{aligned} S(u,v) = au^2 + 2buv + cv^2 \end{aligned}$$

These fit constants (*a,b,c*) are then used to calculate the principal curvatures $k_1$ and $k_2$ (Eq. 4) which are the maximum and minimum rates of curvature at each point.

4 $$\begin{aligned} k_1 = a + c + \sqrt{(a - c)^2 + 4b^2}, \quad k_2 = a + c - \sqrt{(a - c)^2 + 4b^2} \end{aligned}$$

We then compute mean curvature measures using $M = \frac{1}{2}(k_1 + k_2)$. Finally, we assess curvature changes by comparing the baseline and follow-up measurements as it is a useful marker for ventricular shape changes.

*Ventricular surface area stretch*      We define surface area stretch, $\lambda _{\text {A}}$, as the relative surface area change. For each element, we compute the fraction between deformed (surface area at follow-up) and undeformed (surface area at baseline) configuration given by the following expression

5 $$\begin{aligned} \lambda _{{A}} = \frac{A_{\text {deformed}}}{A_{\text {undeformed}}}. \end{aligned}$$

To obtain a smoothed area stretch field, we calculate an area-weighted average from neighboring elements for each node.

*Maximum principal wall strain*      The ventricular surface undergoes pronounced deformation during aging and strain is a suitable measure to quantify local loading of tissue structures. To that end, we compute the maximum principal wall strain across the ventricular surface. As such, we first construct tensors $\textbf{X}$ and $\textbf{x}$ which span the edges of a triangle in the undeformed and deformed configurations, respectively. Specifically, we define $\textbf{X}=[X_2-X_1, X_3-X_1]$ and $\textbf{x}=[x_2-x_1, x_3-x_1]$, where $\textbf{X}_i$ and $\textbf{x}_j$ with $i, j\,=\, 1,\,2,\,3$ are a single triangle’s nodal coordinates in the reference and deformed configuration, respectively. From classic continuum mechanics theory, we know that the mapping between both configurations, also known as the deformation gradient $\textbf{F}$, is given by (Holzapfel 2002)

$$\begin{aligned} \textbf{F}\,=\,\textbf{x}\cdot \textbf{X}^{-1}. \end{aligned}$$

From the deformation gradient we can compute the Green-Lagrange strain tensor $\textbf{E}$ as follows

$$\begin{aligned} \textbf{E}=1/2\,(\,\textbf{F}^T\textbf{F}\,-\,\textbf{I}\,), \end{aligned}$$

with the identity tensor $\textbf{I}$. Lastly, we define the maximum principal wall strain as the largest eigenvalue of strain tensor $\textbf{E}$ by solving the characteristic equation

$$\begin{aligned} det(\,\textbf{E}\,-\,\lambda \,\textbf{I}\,)\,=\,0, \end{aligned}$$

with principal strains $\lambda _i$.

### Periventricular white matter hyperintensity quantification and probability mapping

For the 39 subjects with two FLAIR images, we determine periventricular white matter hyperintensity locations by interpolating their personalized ventricular surface mesh onto their co-registered FLAIR baseline and follow-up images and store the image intensity value at each node. For each subject, we define their ventricular white matter hyperintensity locations wherever the nodal intensity value exceeds their 90th percentile baseline FLAIR intensity value. Longitudinal growth was measured by applying the same threshold to follow-up intensities to see where new white matter hyperintensities emerged.

We obtain a cohort-level representation of white matter hyperintensity distribution by constructing a surface-based probability map. For each surface node *i*, we computed the fraction of subjects exhibiting white matter hyperintensity at that location,

6 $$\begin{aligned} P_i = \frac{1}{N} \sum _{s=1}^{N} W_i^{(s)}, \end{aligned}$$

where $W_i^{(s)} \in \{0,1\}$ indicates whether subject *s* exhibited a white matter hyperintensity at node *i*, and *N* is the total number of subjects. Longitudinal probability change was computed as the node-wise difference between follow-up, $P_i^{\text {fu}}$, and baseline, $P_i^{\text {bl}}$, i.e.,

7 $$\begin{aligned} \Delta P_i = P_i^{\text {fu}} - P_i^{\text {bl}}. \end{aligned}$$

### Statistical analysis of ventricular mechanomarkers and white matter hyperintensity fields

To evaluate differences between female and male subjects, statistical analyses were performed using MATLAB. For scalar measures, sex differences were evaluated via independent t-tests with FDR correction at the cohort level. Correspondingly, mechanomarkers, such as the displacement magnitude, curvature, area stretch, and maximum principal wall strain, were performed via an independent t-test at each node of our ventricular surface mesh. To determine hemispheric differences, i.e., uniformity of the ventricular deformation field, we determined a node-wise mapping between both hemispheres and performed independent t-tests at each node, as outlined in the supplementary materials. Lastly, we statistically analyze the variance of our markers across the cohort to determine which measures to determine homogeneity across our cohort. To that end, we computed the Kullback–Leibler divergence between two variance fields of each mechanomarker; we refer to the supplementary materials for additional information. A significance threshold of *p* < 0.05 was used to determine statistically significant differences. All t-tests assumed unequal variances.

To investigate the relationship between mechanical loading and white matter hyperintensity burden, each ventricular surface mesh node was binned based on white matter hyperintensity probability into groups called "no WMH" (0%), "low" (<33%), "intermediate" (33–66%), and "high" (>66%). For each subject, the average mechanomarker value was computed across nodes belonging to each probability bin. Ventricular nodes adjacent to deep grey matter structures were excluded to avoid misclassification of white matter hyperintensities in non-white matter regions. Group differences across white matter hyperintensity probability bins were assessed using one-way analysis of variance (ANOVA). Effect sizes were quantified using $\eta ^2$. When significant main effects were observed, post hoc pairwise comparisons were performed between bins.

## Results

### Registration accuracy with respect to Dice score, HD, and ASSD

Based on our proposed registration approach, we observe that the Dice score for the whole brain increases from 0.87 ± 0.09 for the raw data to 0.92 ± 0.06 after all registration steps; for the lateral ventricles specifically, we observe an increase from 0.45 ± 0.24 to 0.66 ± 0.25. That corresponds to an average overall improvement of 5.80 ± 12.43% for the full brain and 48.10 ± 42.01% for the lateral ventricles. To further assess registration accuracy, we computed Hausdorff distance (HD) and the Average Symmetric Surface Distance (ASSD). At the full brain level, we found that HD decreased from 21.03 ± 9.37 mm for the raw data to 19.11 ± 9.48 mm after all registration steps; for the ventricles, HD decreased from 18.64 ± 11.10 mm to 16.07 ± 11.42 mm. That corresponds to an improvement for the full brain of 10.95 ± 33.17% and 15.38 ± 16.11% for the ventricle, respectively. ASSD decreased from 4.80 ± 3.36 mm to 2.74 ± 2.68 mm at the full brain level and from 4.30 ± 4.69 mm to 3.34 ± 5.09 mm for the lateral ventricles specifically. That corresponds to an improvement for the full brain of 42.92 ± 23.14% and 22.33 ± 4.19% for the ventricle, respectively. We refer to Fig. S3 in the supplementary materials for visual representation of these findings. While ventricle Dice scores remain relatively low, the statistically significant improvements observed across Dice, HD, and ASSD (p < 0.05) indicate a meaningful enhancement in spatial correspondence between baseline and follow-up scans. We would also like to point out that the registration steps presented here could be performed using a different registration algorithm, such as SyN from the ANTs toolkit (Avants et al. 2011) or MMORF from FSL (Lange et al. 2024) for example, if better suited for another study’s cohort. To further contextualize our registration results, we evaluated a subset of 10 randomly selected subjects using both FSL and ANTs SyN. For one, we observed that FSL took 30 min per subject, while ANTs took 60 min per subject. For the other, as summarized in the supplementary materials, ANTs exhibited reduced variance in surface alignment at the whole-brain level, though this was not consistent across all metrics or regions. For example, FSL showed lower variability in ventricle-level HD, while mean HD values across methods remained comparable. These findings underscore that registration algorithm choice can influence subject-level consistency in a metric- and region-dependent manner, which is particularly relevant for surface-based analyses like the present study. Despite this, our evaluation indicates that FSL provides sufficient accuracy to support reliable surface-based displacement analysis over time. In our comparison, FSL showed a higher Dice score (p < 0.05) but also a higher HD (p < 0.001) and ASSD (p < 0.001) compared to ANTs. Dice score, HD, and ASSD changed from 0.90 ± 0.04 to 0.88 ± 0.04, from 20.96 ± 7.17 mm to 12.31 ± 9.73 mm, and from 3.59 ± 1.29 mm to 1.37 ± 0.47 mm respectively. At the ventricle level, however, the differences were not significant with Dice score, HD, and ASSD changing from 0.54 ± 0.17 to 0.78 ± 0.12, from 10.96 ± 2.97 mm to 12.45 ± 6.95 mm and from 2.52 ± 1.03 mm to 0.89 ± 0.34 mm, respectively. We refer the reader to Figs. S4 and S5 in the supplementary materials for additional outcomes from our comparison between ANTs and FSL with respect to the ventricular deformation fields. Ultimately, we chose FSL for its seamless integration in our overall image processing pipeline.

The lateral ventricular wall represents a geometrically complex and high-contrast boundary between cerebrospinal fluid and parenchyma, making it particularly sensitive to small mismatches between the deformation model and subject-specific anatomy. The mechanomarkers in our analysis are derived from the nonlinear registration field, with area stretch and maximum principal wall strain based on first-order spatial derivatives of the displacement field and curvature based on a second-order derivative. Therefore, these metrics inherit uncertainty from the registration step, with higher-order derivatives expected to be more sensitive to local registration errors. In addition, applying identical registration parameters across the entire cohort may introduce localized discrepancies due to subject-specific anatomical variability. FNIRT employs a spline-based deformation model with bending-energy regularization (Jenkinson et al. 2012; Jenkinson and Smith 2001), which enforces spatial smoothness and suppresses small-scale fluctuations in the displacement field. In our implementation, the regularization parameter (–ssqlambda=1) was kept at its default value, scaling the regularization term relative to the image mismatch and maintaining a stable balance between alignment accuracy and deformation smoothness (Andersson et al. 2010; Jenkinson et al. 2012). This regularization limits the propagation of voxel-level noise and local misregistration into derivative-based metrics such as strain and curvature. Consistent with this, displacement, area stretch, and maximum principal wall strain remained stable across mesh resolutions, while curvature exhibited greater sensitivity, as expected from its second-order dependence on the deformation field. More generally, it is important to keep in mind that registration algorithms involve a trade-off between maximizing spatial overlap and regularizing the underlying warp field. Strong regularization promotes smoothness at the cost of alignment accuracy, whereas weaker regularization may improve overlap metrics (e.g., Dice, Hausdorff distance) but introduce anatomically implausible deformations based on nonlinear elasticity theory. As with any deformable registration, some portion of the measured displacement likely reflects residual alignment uncertainty, especially near boundaries. Future work should address this limitation by incorporating physics-based material models (e.g., hyperelastic models based on nonlinear elastic theory) to adequately constrains the warp field to realistic brain deformations (Burger et al. 2013; Amiri-Hezaveh et al. 2025). Nonetheless, the improvements observed across Dice, HD, and ASSD support the suitability of using FSL in the registration pipeline for extracting anatomical changes (rather than registration errors) from longitudinal imaging of the present cohort.

### Volumetric and surface area change

**Fig. 2:**
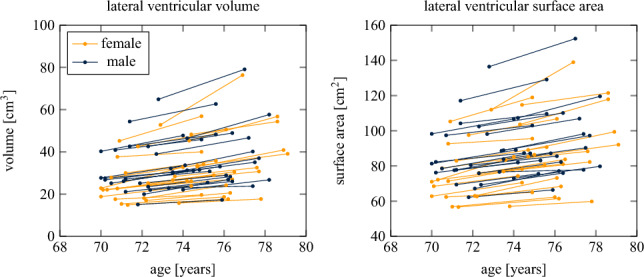
Lateral ventricular volume and surface area change between baseline and follow-up across our cohort. Volume increases on average by 20.1% and surface area increases on average by 9.4% across our 4–5 year observation period, respectively. We observe no statistically significant difference based on sex

Figure 2 shows our cohort’s age-related volume and surface area change over the 4–5 year observation period. Overall, lateral ventricular volume was on average 30.06 ± 11.55 cm$^3$ at baseline and increased on average by 20.1% or 6.05 ± 3.69 cm$^3$ (6.19 ± 4.49 cm$^3$ female; 5.92 ± 2.75 cm$^3$ male) over the 4–5 year observation period. Interestingly, the left hemisphere’s volume expansion rate of 3.08 ± 1.70 cm$^3$ is marginally larger than the right hemisphere’s of 2.98 ± 2.06 cm$^3$ (p = 0.38). Average lateral ventricular surface area was 82.97 ± 17.57 cm$^2$ at baseline and increased by 9.4 % or 7.85 ± 4.15 cm$^2$ (8.16 ± 5.09 cm$^2$ female; 7.55 ± 3.01 cm$^2$ male) by the time of their follow-up scan. We generally observe that the larger the ventricular volume at baseline, the larger is its increase by the end of the observation period; the same holds true for the ventricular surface area. That is reflected in a Pearson correlation coefficient for volume change of 0.69 (*p* < 0.001); the correlation coefficient for surface area change is 0.54 (p < 0.001). While we see a clear increase in both measures across our cohort, we do not observe any sex-based differences for either measure.

### Ventricular deformation

**Fig. 3:**
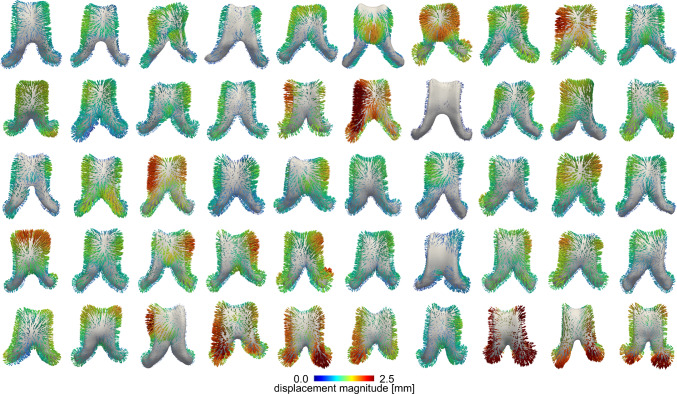
Displacement vector field color coded by displacement magnitude across all 50 subjects. We observed a mostly uniform expansion of the lateral ventricle with a consistent localization of maximum displacements along the edges of the main body. The average displacement magnitude across all subjects is 0.88 ± 0.3 mm (0.92 ± 0.35 mm female; 0.83 ± 0.24 mm male). Vectors are plotted for every 10th node of the ventricular surface mesh and scaled by a factor of 8. Subjects are ordered by ventricular volume at baseline

Figure 3 shows the ventricular displacement field of every subject sorted by ventricular volume at baseline. Across all subjects, we generally observe a predominant expansion of the ventricles with an average displacement magnitude of 0.88 ± 0.46 mm (0.92 ± 0.50 mm female; 0.83 ± 0.41 mm male). Maximum displacements consistently localize along the edges of the main body with an average maximum displacement magnitude of 1.8 ± 0.7 mm and average minimal displacement magnitude of 0.14 ± 0.11 mm. These extreme values indicate that ventricular deformations across our 4–5 year observation period are on the order of our images’ spatial resolution. This provides insight into potential limitations of registration methods to detect brain shape changes on shorter observation periods. We confirmed that our registration framework provides anatomically reasonable deformation fields by computing full brain and ventricle-specific dice scores as well as Hausdorff distance and averaged symmetric surface distance error measures, as reported in the previous section. Overall, our data suggests that the ventricle’s main body undergoes a mostly uniform and symmetric displacement, while the edges show largest displacement magnitudes and anterior horns undergo large radial expansion. Comparison between subjects show very few instances of asymmetric deformation patterns between the left and right ventricle and no sex-based differences are observed. To verify uniformity and symmetry, we quantified node-wise hemispheric differences and report both relative differences and statistically significant differences between the left and right hemisphere as outlined in the supplementary materials, see Fig. S6.

### Mechanical loading of the ventricular wall

**Fig. 4:**
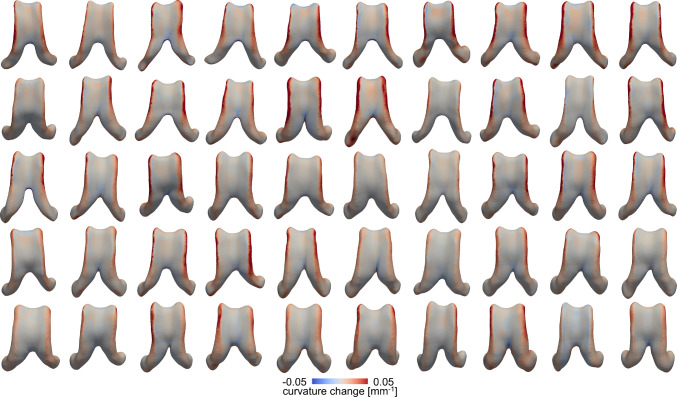
Ventricular curvature change for all subjects. A negative curvature change is associated with flattening and localizes mostly along the edges of the ventricle’s main body. The average curvature change is −0.008 ± 0.003 1/mm across all subjects (−0.008 ± 0.003 1/mm across all female subjects and −0.007 ± 0.003 1/mm across all male subjects)

Figure 4 shows curvature change of the lateral ventricular surface after our 4–5 year observation period. The average curvature change is −0.008 ± 0.015 1/mm across all subjects (−0.008 ± 0.015 1/mm female; −0.007 ± 0.014 1/mm male). A negative curvature change indicates flattening of the surface, while a positive curvature change is associated with an increased bending of the wall. Decreasing curvature change consistently localizes along the edges of the ventricular wall and in the ventricle’s anterior horns. The ventricle’s main body shows marginal changes which suggest that the surface does not change its shape very much. Notably, wall displacement magnitude does not affect the spatial distribution of curvature change. The only impact of displacement magnitude that we observe is the extent to which the edge of the ventricular wall deforms in comparison to the main body.

**Fig. 5:**
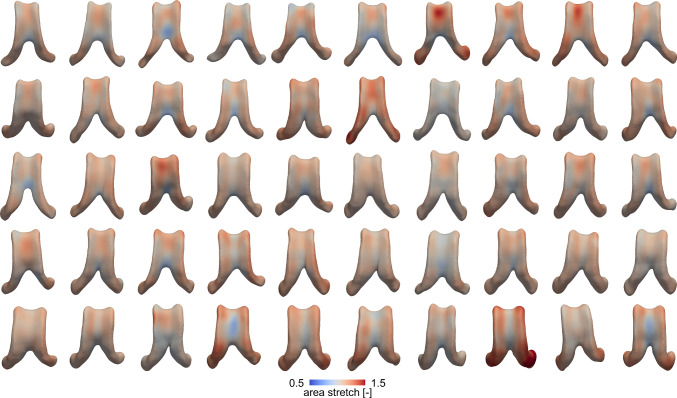
Ventricular area stretch of each subject derived from their ventricular deformations resulting from 4–5 years of aging. Area stretch is highest in the anterior horns and consistently low across most of the ventricle’s main body. Average area stretch is 1.09 ± 0.073 [-] (1.10 ± 0.081 [-] for all female subjects and 1.08 ± 0.06 [-] for all male subjects)

Figure 5 shows each subject’s ventricular surface area stretch after 4–5 years of aging. The average area stretch across all subjects is 1.09 ± 0.073 [-] (1.10 ± 0.081 [-] female; 1.08 ± 0.06 [-] male). This suggests a 9% increase in area within 4–5 years. We notice that subjects with high wall displacements inherently have higher area stretches across the ventricular wall. Additionally, we consistently observe lowest area stretch within the ventricle’s main body, highest area stretch in the anterior horn, and slightly lower area stretches in the posterior horn. Understanding the specific locations that undergo high stretch is important for identifying ventricular regions particularly vulnerable to damage.

**Fig. 6:**
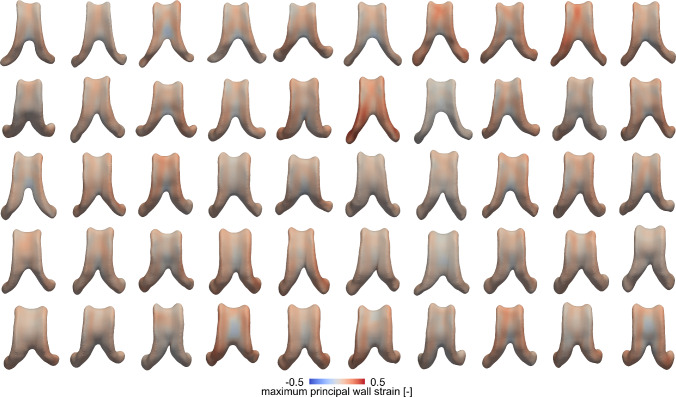
Maximum principal wall strain across the ventricular surface during our 4–5 year observation period. We observe a frequent localization of peak maximum principal wall strain along the edges of the ventricle’s main body and the ventricle’s atrium. The average maximum principal wall strain is 0.091±0.052 [-] (0.098 ±0.057 [-] across all female subjects and 0.085±0.046 [-] across all male subjects)

Figure 6 shows the maximum principal strain across the ventricular surface after 4–5 years of ventricular expansion. In most subjects, highest strains are found along the edges of the ventricle’s main body. In some subjects, the top of the main body experiences elevated positive strain as well which corresponds to overall tensile loads on the cells forming this functional barrier. Only few regions, i.e., mostly the posterior end of the main body as well as the bottom surface experience negative strain or compression. Overall, the average maximum principal wall strain is 0.091 ± 0.052 [-] (0.098 ± 0.057 [-] female; 0.085 ± 0.046 [-] male).

### Summary of our mechanomarkers across our cohort

**Fig. 7:**
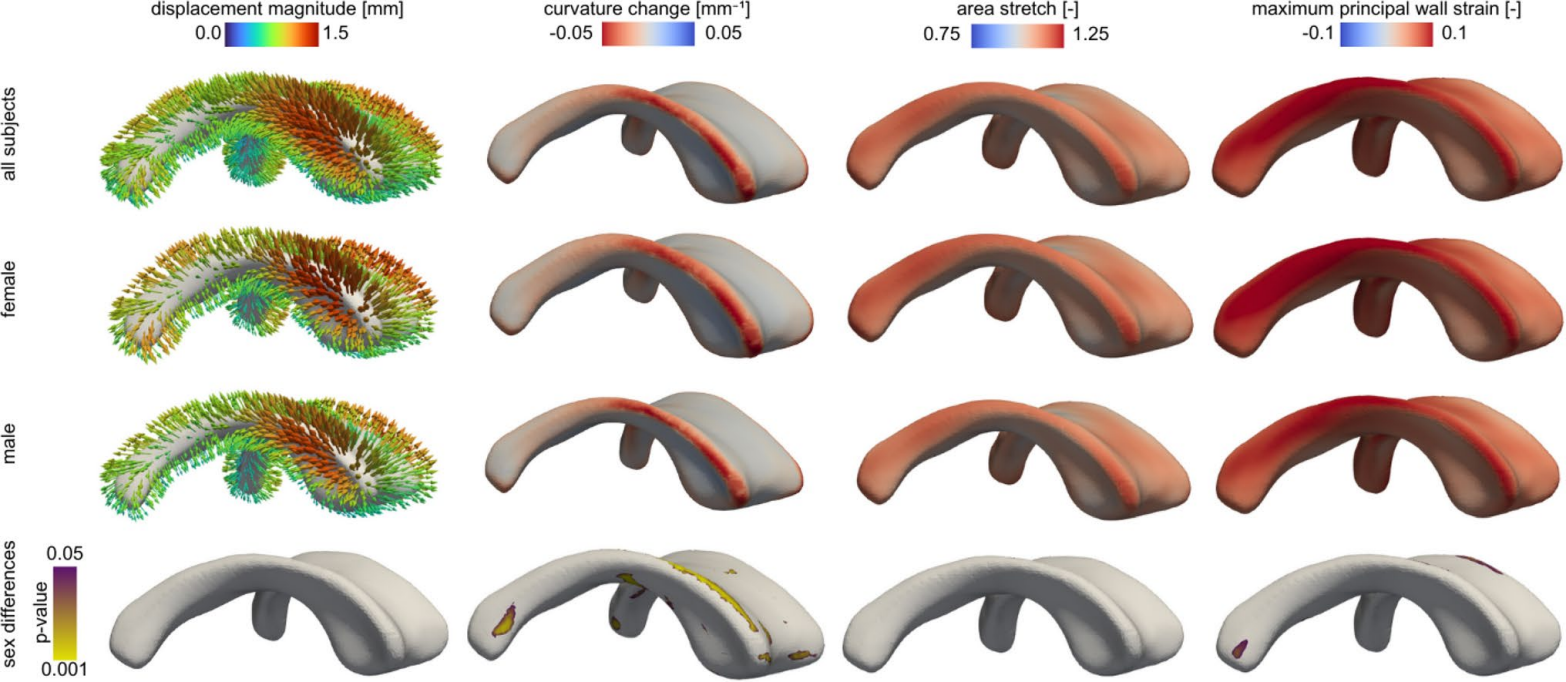
Summary of mechanomarkers across the cohort showing averaged displacement field, averaged area stretch, averaged maximum principal wall strain, and averaged curvature change across the entire cohort (top row), all female subjects (second row), and all male subjects (third row). We also plot the p-value field indicating where sex differences are statistically significant (forth row). There are minimal locations where sex differences are significant

Figure 7 shows a summary of our mechanomarkers across our cohort and average based on sex. Main observations include i) a high level of symmetry between the left and right ventricular hemispheres, ii) no statistically significant difference between female and male subjects across nearly the entire ventricular surface, and iii) consistent localization of our mechanomarkers in regions commonly associated with periventricular white matter injury and the onset of periventricular white matter hyperintensities. The absolute maximum displacement of the averaged field is 1.28 mm (1.32 mm female; 1.24 mm; male). The maximum negative curvature change, i.e., a measure of surface flattening, in our averaged cohort is −0.17 1/mm (−0.17 1/mm female; −0.18 1/mm male). Maximum principal wall strain, i.e., a measure of in-place mechanical loading of the wall, is 0.091±0.052 [-] (0.098 ±0.057 [-] female; 0.085±0.046 [-] male).

Among all measures, the curvature change field is the most consistent field across all subjects. To verify that observation, we compared the variance fields on each normalized marker and computed the Kullback–Leibler divergence, a measure to quantify the similarity between two probability distributions. As outlined in the supplementary materials, we first compare variance fields (see Fig. S7a) and observe that curvature is (i) the most homogeneous field across our cohort and (ii) that the node-wise variance field for curvature varies least in comparison to all other markers. This is further reflected in highest Kullback–Leibler divergence values as shown in Fig. S8 and summarized in Table S1 in the supplementary materials. Measure specific observations include i) higher displacements in the anterior section of the main body’s edges, ii) greater area stretch in anterior and posterior horns, iii) peak maximum principal wall strain along the edges of the ventricle’s main body and the atrium, and (iv) practically no statistically significant differences between female and male subjects for any of the four fields.

**Fig. 8:**
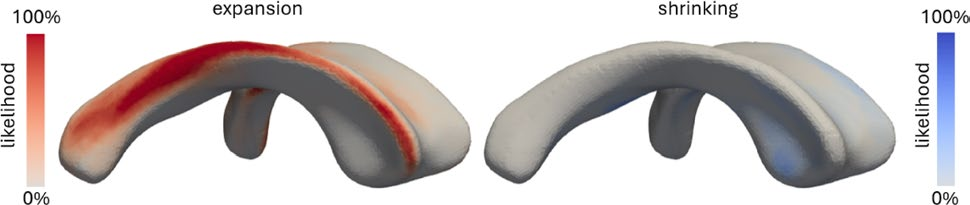
Likelihood that the lateral ventricle will experience peak mechanical loads associated with either **a** wall expansion, i.e., surface area increases, or **b** wall shrinking, i.e., surface area decreases. anterior horns, edge of the ventricle’s main body, and atrium are most likely to expand while the ventricle’s main body has low likelihood to shrink

Figure 8 shows the likelihood that the lateral ventricle experiences peak mechanical loads that are associated with either expansion or shrinking. For each subject, we identified all nodes for which (i) area stretch is >1 and maximum principal wall strain is above the 90th percentile and (ii) area stretch is <1 and maximum principal wall strain is below the 10th percentile. For each node, we then divide the total number of subjects for which either criterion is satisfied and divide it by the total number of subjects. It is evident from the figure that the edges of the ventricle’s main body as well as the atrium are most likely to experience wall expansion while there is only marginal likelihood that the ventricular wall will shrink in any location across the ventricular surface. As such, we observe that 29.2 ± 9.3 % of the ventricular wall may experience maximum mechanical loading associated with expansion and 4.4 ±2.5 % of the ventricular wall experience peak negative mechanical loading associated with shrinking. Most of the other wall sections may expand but experience lower wall strain levels.

### Mechanomarker sensitivity to ventricular surface mesh resolution

**Fig. 9:**
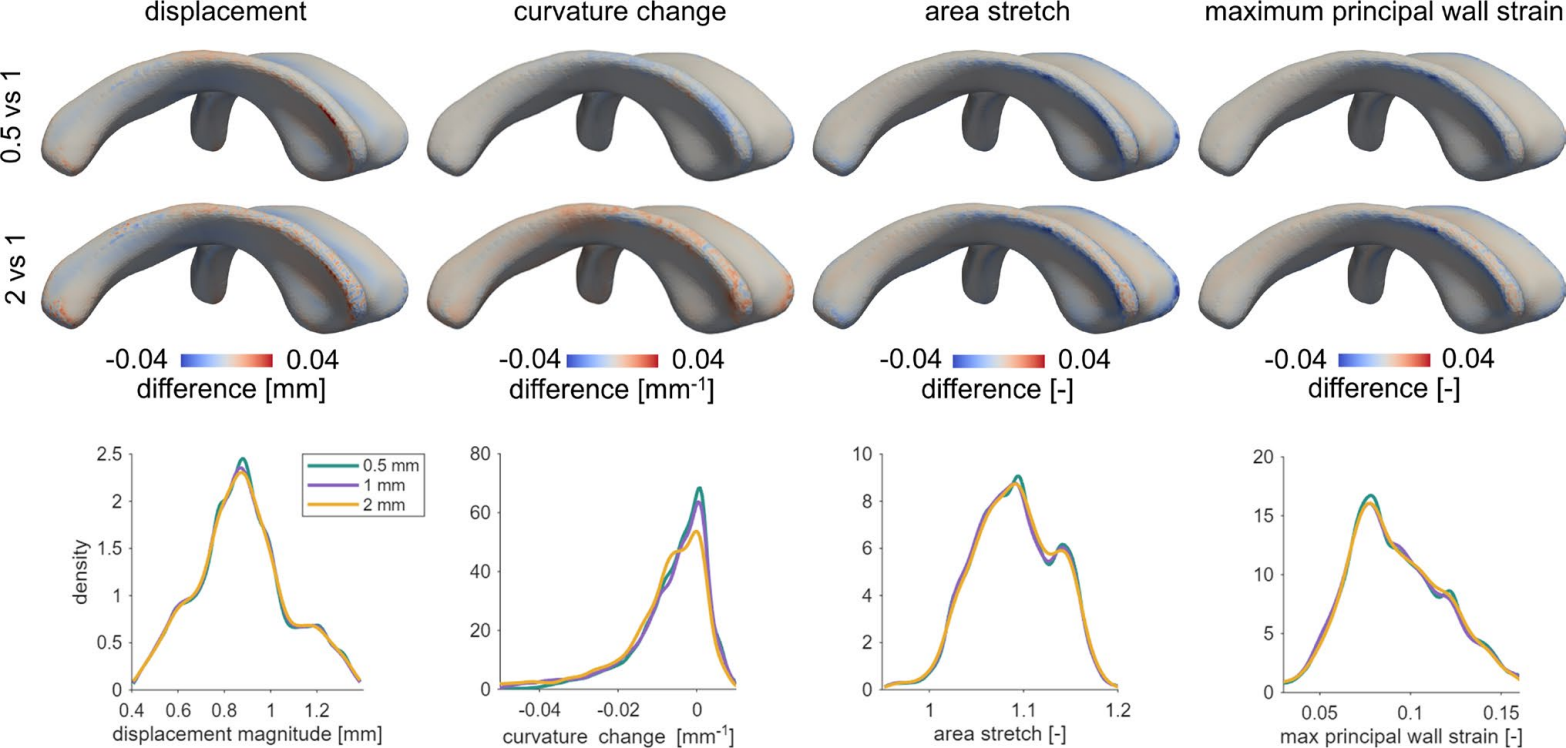
Mesh resolution sensitivity analysis. Top: spatial difference maps of displacement, curvature change, area stretch, and maximum principal wall strain after mapping the 0.5 mm and 2 mm meshes onto the 1 mm reference mesh. Differences are primarily localized to boundary regions (edges, anterior horns, and atrium), while the main body remains largely unchanged. Curvature change shows the greatest sensitivity to mesh resolution, with reduced values for the 0.5 mm mesh and increased values for the 2 mm mesh, whereas area stretch and maximum principal wall strain show consistently lower values relative to the 1 mm mesh. Bottom: histogram comparisons showing strong agreement in distribution shape across resolutions, with differences mainly reflected in peak density. The 0.5 mm mesh shows higher peak concentrations, while the 2 mm mesh shows broader distributions. Overall, results indicate localized spatial differences and preserved global distributions across mesh resolutions

Our mechanomarkers are derived from the nonlinear registration field with area stretch and maximum principal wall strain derived from first-order spatial derivatives and curvature change derived from a second-order derivative of the displacement field. Therefore, these quantities inherit any uncertainty from the registration step, with higher-order derivatives expected to be more sensitive to local errors. To assess the sensitivity of the computed deformation metrics to mesh resolution, we performed a multi-resolution analysis of our mesh with a prescribed 0.5 mm, 1 mm, and 2 mm average element edge length, respectively. The 1 mm mesh was used as the reference model as it corresponds to the MRI voxel resolution. For each resolution, we independently ran our registration steps to determine displacement magnitude, curvature change, area stretch, and maximum principal wall strain. The 0.5 mm and 2 mm results were mapped onto the 1 mm mesh using MATLAB’s scattered interpolation function, enabling point-wise comparison across resolutions. Resolution sensitivity was evaluated using the normalized root mean square error (NRMSE) relative to the 1 mm mesh, spatial difference maps, and distributional comparisons via histograms. As summarized in Table 2, displacement exhibited NRMSE values of 0.0052 (0.5 vs 1 mm) and 0.0069 (2 vs 1 mm), while area stretch showed errors of 0.0041 and 0.0051, respectively. Maximum principal wall strain demonstrated similarly low sensitivity, with NRMSE values of 0.0372 and 0.0470. In contrast, curvature change exhibited higher NRMSE values (0.2444 and 0.3594, respectively), consistent with its dependence on second-order spatial derivatives and the known resolution sensitivity of curvature-based quantities. As shown in the top two rows of Fig. 9, spatial difference maps show only localized differences. Across all measures, for example, difference maps show higher positive differences concentrated at the edges, anterior horn, and atrium, while the main body shows predominantly very small negative differences. This indicates that mesh resolution primarily affects regions with higher curvature, whereas the central–rather flat–regions, such as the main body, are much less unaffected. We generally observe that the 2 mm mesh exhibits more pronounced spatially heterogeneous differences compared to the 0.5 mm mesh which shows more homogeneously distributed differences with respect to the template mesh. Histogram comparisons shown in the bottom row of Fig. 9, demonstrate strong agreement across mesh resolutions, with differences mostly reflected in peak density rather than distribution shape. For displacement, the 0.5 mm shows the highest peak at 0.88 mm, indicating a greater concentration of nodes at this value, while the 1 mm and 2 mm meshes show slightly lower peak densities but with similar distribution spread. Curvature change shows the most noticeable variation between resolutions. The 0.5 mm and 1 mm mesh maintain similar distribution shapes, with the finer mesh showing a higher peak density at -9E-5 mm$^{-1}$. In contrast, the 2 mm mesh displays are broader distribution with a lower peak and a more skewed distribution curve, suggesting increased variability and reduced consistency in curvature change approximations at the coarser resolution. For area stretch, all three meshes follow a similar distribution up to the first peak. The 0.5 mm mesh shows a slightly higher peak at 1.09, indicating increased concentration of nodes with that value. Following the first peak, the 2 mm mesh shows a less pronounced drop in density before the secondary peak and does not decrease as sharply as the finer meshes. Maximum principal wall strain shows highly consistent distribution shapes across all resolutions, with mesh densities peaking at 0.07. The 0.5 mm mesh again demonstrates a slightly higher peak density. Overall, the differences are minimal and limited to changes in density rather than shifts in value range.

**Table 2:** Normalized root mean square error (NRMSE) for each mechanomarker after mapping the 0.5 mm and 2 mm meshes onto the 1 mm reference mesh. Displacement, area stretch, and maximum principal wall strain exhibit low sensitivity to mesh resolution, while curvature change shows higher error consistent with its dependence on second-order spatial derivatives

	Displacement	Curvature change	Area stretch	Maximum principal wall strain
0.5 vs 1	0.0052	0.2444	0.0041	0.0372
2 vs 1	0.0069	0.3594	0.0051	0.0470

### White matter hyperintensities distribution

**Fig. 10:**
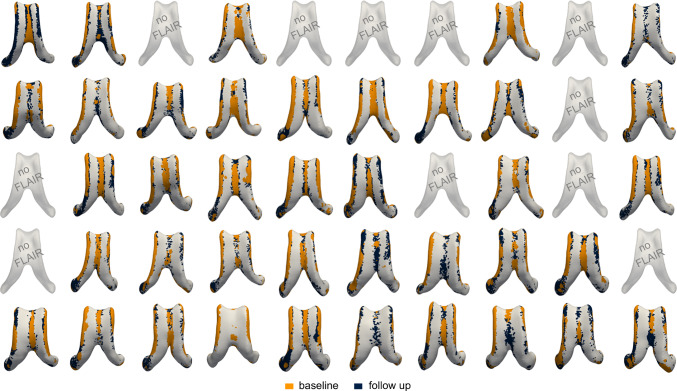
Subject white matter hyperintensity distributions at baseline (orange) and follow-up (blue). White matter hyperintensities consistently localize to the anterior horns and ventricular edges and show growth over the 5-year observation period

Figure 10 illustrates the spatial distribution of periventricular white matter hyperintensities across all subjects at baseline and follow-up. White matter hyperintensities predominantly localize along the edges of the ventricular body and in the anterior and posterior horns. We observe a consistent pattern of white matter hyperintensity expansion over the 5-year observation period. In the subject with the lowest ventricular baseline volume, white matter hyperintensities at baseline are localized to the anterior horns and near the septum. At follow-up, these lesions extend outward from their initial locations and grow along the ventricular wall. A similar pattern is observed in the subject with the largest baseline ventricular volume. Despite the larger ventricular geometry, white matter hyperintensities again localize to the anterior horns and wall at baseline and subsequently expand along the ventricular edges at follow-up. These observations suggest that the spatial pattern of white matter hyperintensity growth is broadly consistent across subjects with varying levels of ventricular enlargement.

**Fig. 11:**
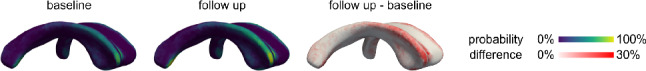
Cohort-averaged white matter hyperintensity probability maps at baseline and follow-up. The difference map highlights regions of white matter hyperintensity probability increase over time, showing an up to 30% increase in probability, primarily localized to the anterior horns and ventricular edges

Figure 11 shows cohort-averaged white matter hyperintensity probability maps at baseline and follow-up. At baseline, white matter hyperintensity probability is highest along the anterior horns and lateral edges of the ventricular body. At follow-up, these regions extend further along the edges of the ventricular horns and atrium whole the ventricle’s main body experiences no significant change. The difference map between follow-up and baseline highlights this progression pattern, showing localized increases of up to $30\%$ around the anterior horns and along the ventricular edges. In contrast, the ventricular body exhibits comparatively low baseline white matter hyperintensity probability and minimal longitudinal change. Overall, these observations indicate that white matter hyperintensity burden progresses primarily along the ventricular edges and anterior horns, with longitudinal growth extending outward from pre-existing lesion locations.

To assess whether ventricular mechanics differ in regions associated with white matter hyperintensity burden, mechanomarkers were compared between nodes with and without white matter hyperintensity. Across the cohort, nodes associated with white matter hyperintensity exhibited slightly larger displacement magnitudes ($0.91\pm 0.37$ mm) compared to non-white matter hyperintensity nodes ($0.88\pm 0.38$ mm), with this difference reaching statistical significance ($p<0.01$). Similarly, curvature change is more negative in white matter hyperintensity regions ($-0.01\pm 0.00$ mm$^{-1}$) compared to regions without white matter hyperintensity ($0.00\pm 0.00$ mm$^{-1}$), with this difference also reaching statistical significance ($p<0.001$), indicating a greater longitudinal decrease in mean curvature near white matter hyperintensity accumulation. In contrast, average area stretch did not differ between white matter hyperintensity and non-white matter hyperintensity regions (both $1.11\pm 0.05$), although the statistical test still indicated significance ($p<0.001$). Maximum principal wall strain showed a small but statistically significant increase ($p<0.001$) in white matter hyperintensity-associated regions ($0.11\pm 0.04$) compared to non-white matter hyperintensity regions ($0.10\pm 0.04$), indicating slightly elevated local mechanics near white matter hyperintensity regions.

**Fig. 12:**
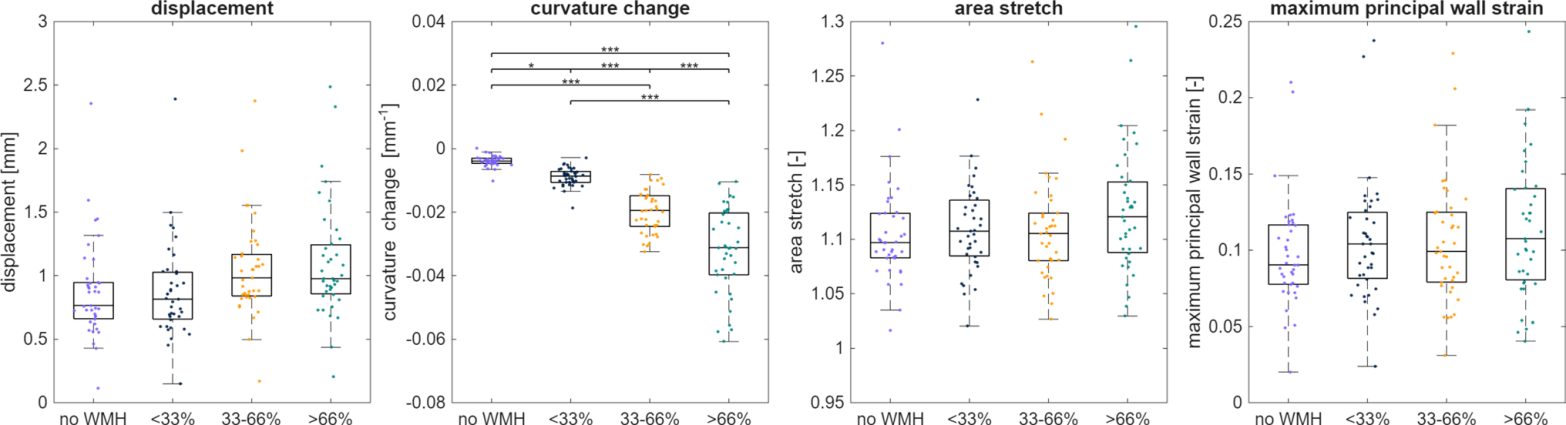
Distribution of mechanomarkers across subjects based on white matter hyperintensity probability bins (no WMH, <33%, 33–66%, >66%). Displacement shows a gradual increase in magnitude from low to high white matter hyperintensity probability regions. Curvature change demonstrates the most pronounced differences between bins, with progressively more negative curvature change observed as white matter hyperintensity probability increases. Area stretch and maximum principal wall strain distributions remain highly similar across probability bins, indicating minimal variation with white matter hyperintensity burden

To further evaluate whether mechanomarkers scale with the magnitude of white matter hyperintensity burden, ventricular nodes were binned into "no WMH", "low" ($<33\%$), "intermediate" (33–$66\%$), or "high" ($>66\%$) probability and averaged on the subject level. Table 3 summarizes the results across the mechanomarkers. Effect size analysis reveals that white matter hyperintensity burden explains a moderate proportion of the variance in curvature change ($\eta ^2=0.69$), while effects on displacement ($\eta ^2=0.06$), area stretch ($\eta ^2=0.03$), and maximum principal wall strain ($\eta ^2=0.03$) are comparatively small. Figure 12 shows the distribution of mechanomarkers across our white matter hyperintensity probability bins. Displacement shows a gradual increase in magnitude from low to high white matter hyperintensity probability regions. Curvature change demonstrates the most pronounced differences between bins, with progressively more negative curvature changes observed as white matter hyperintensity probability increases. In contrast, area stretch and maximum principal wall strain distributions remain highly similar across probability bins, indicating minimal variation with white matter hyperintensity burden.

**Table 3:** Average mechanomarker values across subjects based on white matter hyperintensity probability bins (no WMH, <33%, 33–66%, >66%). Effect size analysis reveals that white matter hyperintensity burden explains a moderate proportion of the variance in curvature change ($\eta ^2=0.69$), while effects on displacement ($\eta ^2=0.06$), area stretch ($\eta ^2=0.03$), and maximum principal wall strain ($\eta ^2=0.03$) are comparatively small

	Displacement	Curvature change	Area stretch	Maximum principal wall strain
no WMH	$0.88\pm 0.38$	$0.00\pm 0.00$	$1.11\pm 0.05$	$0.10\pm 0.04$
< 33%	$0.87\pm 0.38$	$-0.01\pm 0.00$	$1.11\pm 0.05$	$0.10\pm 0.04$
33-66%	$1.05\pm 0.38$	$-0.02\pm 0.01$	$1.11\pm 0.05$	$0.11\pm 0.04$
> 66%	$1.10\pm 0.45$	$-0.03\pm 0.01$	$1.13\pm 0.06$	$0.12\pm 0.05$

## Discussion

### Predominantly uniform expansion of the lateral ventricle during aging

Our results suggest that the lateral ventricles uniformly expand during healthy aging. Although displacement magnitudes vary between subjects, the overall deformation patterns are highly consistent across our cohort of cognitively normal adults aged 70–80 years. The edges along the ventricle’s main body deform most, the ventricle’s atrium deforms less, and the upper and lower surface of the main body deform the least. These overall shape changes are consistent with qualitative descriptions of ventricular changes reported in literature (Ferrarini et al. 2006; Shi et al. 2015; Hartkens et al. 2003; Fjell et al. 2009). Previous studies have been primarily limited to short observation periods of about 1–2 years between scans (Madsen et al. 2013; Dong et al. 2020), such that the reported ventricular deformations are significantly smaller than our values and less representative of longer-term aging.

Our volume and surface area changes for subjects between the age of 70 and 80 agree with morphometry-derived measures (Leung et al. 2013; Fujita et al. 2023; Nestor et al. 2008). Specifically, we report an average volume increase of 6.05 ± 3.69 cm$^3$ (or 4.01 ± 1.57%/year) over our 4–5 year observation period. This expansion rate corresponds to about 1.21 ml/year (mean increase over 5 years) which is below the reported values of 1.29 - 1.72 mL/year from Leung et al. (Leung et al. 2013) and not much larger than the annualized lateral ventricular volume increase of about 2.7 - 3.0 %/year for subjects in their 70 s by Fujita et al. (Fujita et al. 2023). Nestor et al. determined ventricular volume changes of about 1.7% over a 6-month observation period which is likely to correspond to a 3.4%/year growth rate (Nestor et al. 2008). Discrepancies between studies are likely caused by differences in the segmentation of ventricles, age ranges, and registration methods used. While we did not observe a statistically significant difference of volumetric expansion between left and right hemisphere, several studies have reported a left-right asymmetry (Stratchko et al. 2016; Trimarchi et al. 2013; Salerno et al. 1992). What might cause these hemispheric differences, however, remains unclear.

It has been repeatedly established that the ventricle’s frontal region undergoes the most notable shape and size changes with age and disease (Ferrarini et al. 2006; Apostolova et al. 2012), followed by the edges of the main body (Ferrarini et al. 2006; Chou et al. 2008), and the ventricle’s atrium (Ferrarini et al. 2006; Chou et al. 2008; Shi et al. 2015; Apostolova et al. 2012). Others reported that the temporal horns, which are adjacent to the hippocampus, show increased shape changes caused by the accelerated tissue volume loss from neurodegeneration in subjects with Alzheimer’s disease (Thompson et al. 2004; Ferrarini et al. 2006). Another report suggested that ventricular volume changes are most notable in the occipital horn near the atrium (Stratchko et al. 2016). We pose that the stark differences of reported shape changes are associated with age of the subjects, number of years between scans, and state of health. In aggregate, these observations all point towards a mostly uniform expansion of the ventricles with rather minor spatial differences between healthy and diseased brains. It is more likely that neurodegenerative diseases drastically accelerate brain aging and, as such, lead to more pronounced shape changes which exceed growth rates observed in cognitively normal subjects (Coupé et al. 2019; Blinkouskaya et al. 2021).

### Co-localization of maximum ventricular loading and clinically-observed periventricular tissue degeneration

Healthy white matter aging is characterized by lesion formation throughout the cerebrum (Liu et al. 2017). The underlying microstructural changes range from vascular degeneration to substantial tissue loss driven by demyelination and axonal damage (Yeatman et al. 2014). White matter hyperintensities appear as bright regions on T2-weighted fluid-attenuated inversion recovery (FLAIR) magnetic resonance images and, strikingly, are present in nearly every aged brain (Wardlaw et al. 2015). More importantly, while early-stage lesions are only weakly associated with cognitive decline (Wang et al. 2019), increasing white matter hyperintensity burden is an indicator of progressive functional decline (De Groot et al. 2002; Schmidt et al. 2005) and associated with neurodegenerative diseases (Chen et al. 2021; Griffanti et al. 2018). Lesions are typically assessed using the Fazekas scale, which distinguishes between periventricular and deep white matter hyperintensities and grades their severity (Wahlund et al. 2001). They initially emerge near the ventricular horns and extend along the ventricular wall into deeper white matter tissue (Fazekas et al. 1993, 1998). This pattern suggests that periventricular lesions are triggered by deterioration of the ventricular wall and subsequently spread into deeper tissue (Sze et al. 1986; Todd et al. 2018). In our previous work, computational modeling demonstrated that mechanical stretch localizes to the ventricular horns (Caçoilo et al. 2023, 2022) and corresponds spatially with ventricular wall loading and lesion locations (Visser et al. 2021, 2023). Consistent with this, our longitudinal analysis shows that displacement, area stretch, and maximum principal wall strain increase slightly with lesion burden, with higher values in the anterior horns and lower values across the ventricle’s main body. This agreement between modeling predictions and longitudinal measurements supports the interpretation that ventricular enlargement concentrates mechanical loading in anatomically vulnerable regions. Among our measures of mechanical loading, curvature change provides the strongest discrimination between lesion burden groups. This aligns with prior findings that high-curvature regions coincide with elevated ependymal cell stretch (Caçoilo et al. 2022). Here, we show that the horns undergo a negative curvature change during ventricular enlargement, reflecting progressive rounding and an increase in local radius. Notably, regions exhibiting more negative curvature change also correspond to higher lesion probability, consistent with earlier observations linking larger anterior horn radii to increased lesion burden (Visser et al. 2021). These results suggest that ventricular shape changes in the horns are a sensitive indicator of disease progression.

From a biological perspective, the ventricular wall is lined by a single layer of ependymal cells that create a barrier between cerebrospinal fluid and periventricular tissue (Kim et al. 2008; Roales-Bujan et al. 2012). Thinning or disruption of the ependymal lining has been reported in aging and small vessel disease and is associated with periventricular white matter damage (Wardlaw et al. 2003). In our study, regions experiencing the highest mechanical loads, particularly the anterior horns and atrium, also correspond to areas where ventricular wall is most susceptible to thinning. Ventricular expansion induces tensile stress that stretches the wall, a phenomenon highly reflective of pathological observations (Shook et al. 2014; Pena et al. 1999) and well-captured by curvature change. For example, our computational models have demonstrated that maximal ependymal stretch localizes to high-curvature regions such as the horns (Caçoilo et al. 2023). Together, these findings support curvature change as a biologically interpretable marker of evolving geometry that helps explain the mechanical loading experienced by the ependymal lining in vulnerable white matter regions. This clear correlation between pronounced ventricular enlargement and diminished wall integrity strongly supports our claim that ventricular deformation and deformation-derived mechanical markers are suitable markers for white matter injury (Visser et al. 2023; Caçoilo et al. 2023).

The observed spatial heterogeneity in our ventricular deformation likely reflects varying degrees of periventricular tissue damage and degeneration (Nestor et al. 2008; Shook et al. 2014; Sze et al. 1986). To that end, Shook et al. reported expansion in the frontal horns and atrium, accompanied by ependymal gliosis and reduced barrier integrity (Shook et al. 2014). Their histological analyses revealed localized gliosis along the ventricular wall, particularly near the atrium, whereas the ependymal lining remained largely intact near connections to deep grey matter structures such as the caudate and thalamus. Consistent with this, 3D volumetric reconstructions showed pronounced ventricular expansion in the frontal horns, the atrium, and portions of the inferior ventricular body. These spatial patterns closely align with our findings, where peak mechanical loads concentrate near the anterior ventricular regions.

The outward progression of periventricular white matter hyperintensities from the ventricular horns aligns with the spatial distribution of mechanical loading localized in these same regions. Lesions begin as smooth caps near the frontal horns, extend along the ventricular wall, and eventually penetrate deeper white matter (Chen et al. 2021; Schmidt et al. 2011). This expansion is associated with degeneration of surrounding normal-appearing white matter (Maniega et al. 2015; Silbert et al. 2024), promoting local inflammation and damage to structures such as myelin (Maillard et al. 2014; Maniega et al. 2015; Silbert et al. 2024). Taken together, the convergence of mechanical, geometric, and biological evidence suggests that ventricular expansion plays a central role in driving periventricular tissue degeneration during brain aging.

### Limitations

In the present study our sample size consisted of only 50 cognitively normal subjects with a tightly defined age range. This limits our ability to generalize our findings to individuals across a wider age range as well as subjects with mild cognitive impairment and Alzheimer’s disease. Future work should include these additional populations to enable a more comprehensive understanding of ventricular changes across different cognitive states and provide further insights into the progression of cognitive decline with age. Ideally, ventricular shape changes can serve as a sensitive marker for early detection of mild cognitive impairment and Alzheimer’s disease. While we observed consistent spatial correspondence between ventricular mechanomarkers and white matter hyperintensity distributions across subjects, the relatively small cohort size may limit statistical power and sensitivity to inter-subject variability. Replication in larger datasets will be important to confirm the stability of these spatial patterns. Additionally, white matter hyperintensity segmentation were done through intensity based thresholding which provides a consistent estimate of lesion distribution, but may not fully capture complex lesion morphology.

## Conclusions

Our registration approach provided the deformation field of lateral ventricles in cognitively normal subjects aged 70–75 years. We observed mostly uniform expansion of the ventricles. Given the highly curved shape in the ventricle’s horns, the corresponding mechanical loading of the ventricular surface is highly heterogeneous. We observe consistent localization of maximum mechanical loading, i.e., curvature change, surface area stretch, and maximum principal wall strain, in the anterior horns, along the edges of the ventricle’s main body, and in the atrium. Strikingly, these regions of elevated mechanical loading spatially coincide with the most prevalent locations of periventricular white matter hyperintensities. Our white matter hyperintensity analysis further demonstrated that mechanical markers increase with lesion burden and that curvature change provides the strongest discrimination between groups. This suggests that ventricular enlargement is not merely an inconsequential byproduct of neurodegeneration, but rather a driving force behind periventricular white matter lesion formation and resulting cognitive decline. The results presented here support this hypothesis and show that ventricular expansion leads to excessive strains on adjacent tissue, potentially accelerating lesion formation. Understanding the corresponding mechanical forces could inform early intervention strategies and help differentiate normal aging from pathological atrophy before significant white matter damage occurs. Additionally, our approach should be used to study subjects with hydrocephalus and ventriculomegaly in order to understand if brain/ventricle shape is a predictor for increased risk of ventricular wall instability and an accelerated expansion of the ventricular volume with age.

## Supplementary Information

Below is the link to the electronic supplementary material.

Supplementary file 1 (pdf 6163 KB)

## Data Availability

No datasets were generated or analysed during the current study.
