# Supplementary material for: Aging-related lateral ventricular shape changes and corresponding mechanical loading derived from longitudinal image registration
Source: Biomech Model Mechanobiol. 2026 Jun 3;25(3):54. doi: 10.1007/s10237-026-02080-8 (PMC13233658; doi:10.1007/s10237-026-02080-8)
Supplement: Supplementary file 1 — (pdf 6163 KB) [file 10237_2026_2080_MOESM1_ESM.pdf]

Supplementary Material: Aging-Related Lateral Ventricular Shape Changes and Corresponding Mechanical Loading Derived from Longitudinal Image Registration

Lauren Cunniff[1] and Johannes Weickenmeier[1,2,3,*]

[1] Department of Mechanical Engineering, Stevens Institute of Technology, Hoboken, NJ 07030
[2] Department of Engineering Science, University of Oxford, Oxford OX3 7DQ, UK
[3] Podium Institute for Sports Medicine and Technology, University of Oxford, Oxford OX3 7DQ, UK
*Corresponding Author. Email: johannes.weickenmeier@eng.ox.ac.uk

1. Definition of ventricular subregions

Figure S1 shows the label of ventricular subregions that we repeatedly reference throughout our manuscript. As such we primarily differentiation between the (superior and inferior sections of the) main body, the left and right edges of the main body, the atrium, and the anterior horns. Given the ventricle's shape, most of the mechanical measures of ventricular loading as well as characteristic shape change measures localize in distinct regions such as along the edges of the ventricle's main body.

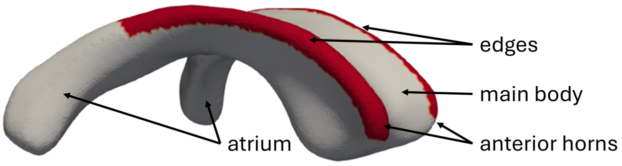

Figure S1: Definition of regional labels of the ventricular surface that are used throughout the manuscript. Specifically, we reference the main body, edges of the main body, atrium, and the anterior horns.

2. Registration accuracy after individual registration steps

Nonlinear registration methods can sometimes provide distorted warp fields that hamper registration results. In order to assess that our proposed workflow leads to satisfactory results, we analyzed full brain and ventricle-specific distance measure errors. As illustrated in Fig. S2, we segmented the ventricles in all baseline images, rigidly aligned follow-up scans, and nonlinearly aligned follow-up scans to quantify the improvement of overlap using our proposed workflow. We then registered the MNI152_T1_2mm_VentricleMask.nii.gz available through FSL to our baseline, the original follow up image, the rigidly-aligned follow-up image (FLIRT), and the nonlinearly-aligned follow-up image (FNIRT).

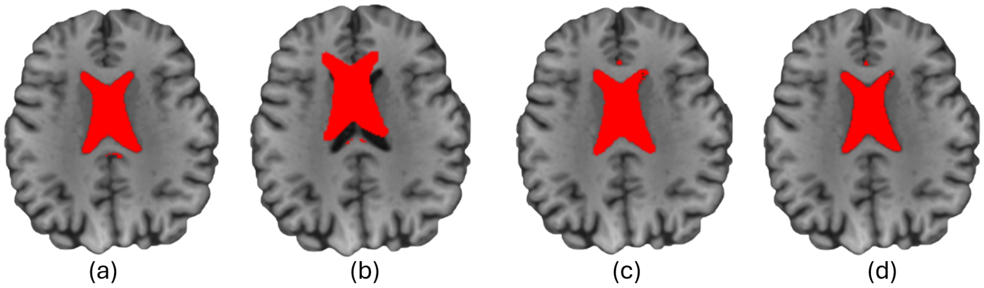

Figure S2: Visualization of ventricle mask alignment across registration steps: (a) Baseline brain with the ventricle mask derived from the baseline image; (b) baseline brain with the ventricle mask from the original follow up image, illustrating the initial misalignment; (c) baseline brain with the ventricle mask from the rigidly aligned follow up image (FLIRT) showing improved alignment; and (d) baseline brain with the ventricle mask from the nonlinearly aligned follow up image (FNIRT), demonstrating further refinement.

To quantify registration accuracy, we computed the full brain and ventricle-specific Dice score, Hausdorff Distance (HD), and Average Symmetric Surface Distance (ASSD) for each subject and present the results in Fig. S3. For the ventricles, we observe reliable alignment after both linear and nonlinear registration, with a 0.22-point improvement in Dice score following linear registration and an additional 0.06-point increase after nonlinear registration. Ventricle surface distance metrics also showed progressive improvement, with HD decreasing by 2.37 mm after FLIRT and by an additional 0.21 mm after FNIRT, and ASSD decreasing by 1.02 mm after FLIRT and by an additional 0.13 mm after FNIRT. When considering the final values after both registration steps (i.e., following FNIRT), Dice scores improved by 5.8 ± 12.4% for the full brain and 48.1 ± 42.0% for the ventricles; HD improved by 11.0 ± 33.2% and 15.4 ± 16.1%, and ASSD improved by 43.0 ± 23.1% and 22.3 ± 4.9%, respectively. The rightmost panels in Fig.S3 summarize the relative percentage improvements for Dice, HD, and ASSD across the cohort. These increases in Dice score and reductions in surface distance metrics support sufficient registration results to extract anatomical changes from longitudinal imaging.

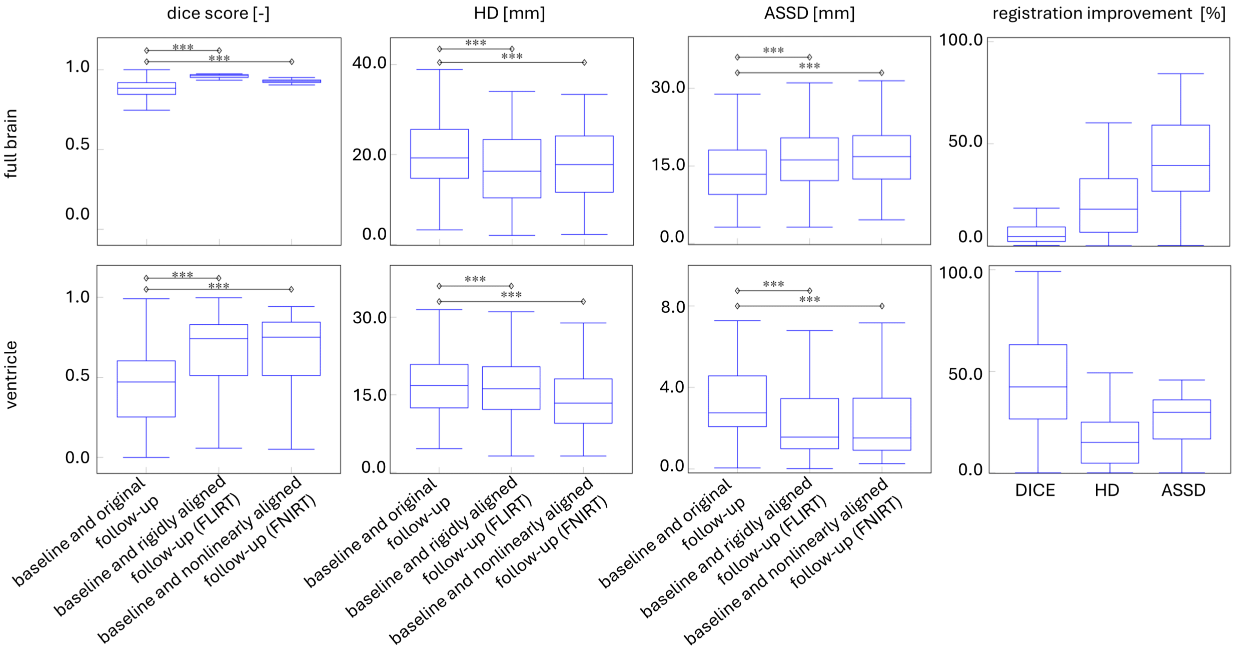

Figure S3: Quantitative assessment of registration accuracy at the full brain and ventricular level. Boxplots illustrate the distribution of Dice score, Hausdorff Distance (HD), and Average Symmetric Surface Distance (ASSD) across subjects at different stages of image registration: baseline vs. original follow-up (unregistered), baseline vs. rigidly aligned follow-up (FLIRT), and baseline vs. nonlinearly aligned follow-up (FNIRT). Higher Dice scores and lower HD and ASSD values indicate better registration performance. Dice scores improved by 5.8 ± 12.4% for the full brain and 48.1 ± 72.0% for the lateral ventricles. HD improved by 11.0 ± 33.2% for the full brain and 15.4 ± 16.1% for the ventricles while ASSD improved by 43.0 ± 23.1% and 22.3 ± 4.9%, respectively. The rightmost panels summarize the relative percentage improvements achieved after registration for each metric. Statistically significant improvements (*** stands for $p < 0.001$) were observed following both rigid and nonlinear registration for full brain and ventricular regions.

2.1. *Registration performance comparison between ANTs and FSL*

To assess the influence of the registration algorithm on registration accuracy, we selected 10 random subjects (5 female, 5 male) and used SyN from the ANTs toolkit to assess if ANTs performs better compared to FSL (which we used in the present study). Specifically, we evaluate registration results with respect to (i) Dice score, HD, and ASSD, and (ii) the resulting ventricular displacement field. Figure S4 summarizes the comparison between both registration methods for the full brain and ventricles, respectively. We observe that at the full brain level, FSL appears to provide a higher Dice score ($p < 0.05$) but also a higher HD ($p < 0.001$) and ASSD ($p < 0.001$) value compared to ANTs. At the ventricle level, we do not see any significant differences but observe that the Dice score ($p = 0.09$) is lower for FSL compared to ANTs while both HD and ASSD are higher with $p = 0.85$ and $p = 0.07$, respectively.

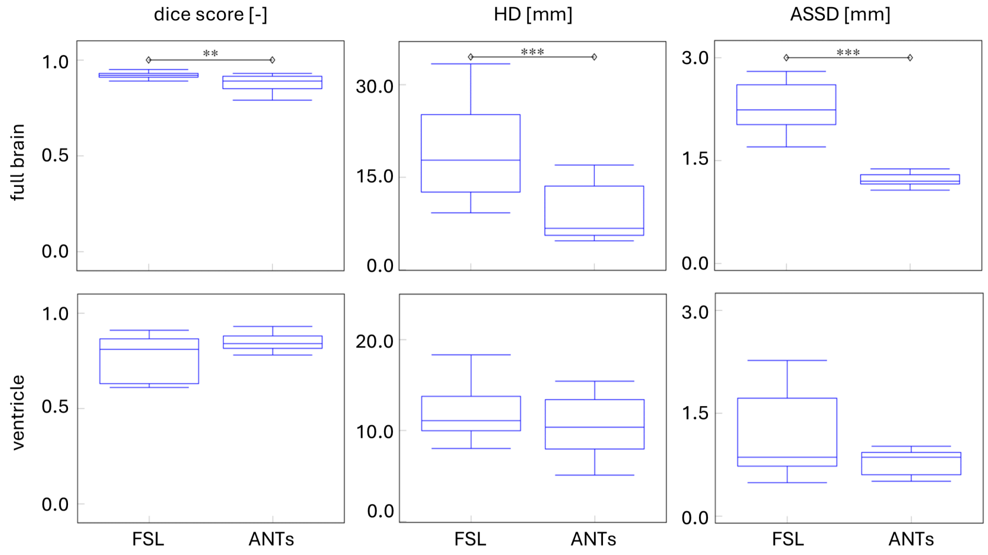

Figure S4: Comparison of registration accuracy between FSL and ANTs registration methods at the full brain and ventricle level. Boxplots show Dice score, Hausdorff Distance (HD), and Average Symmetric Surface Distance (ASSD) across 10 randomly selected subjects (5 female, 5 male). FSL yielded significantly higher Dice scores at the full brain level ($p < 0.05$) but also resulted in significantly higher HD and ASSD values ($p < 0.001$) compared to ANTs. At the ventricular level, no significant differences were observed; however, Dice scores were slightly lower for FSL compared to ANTs ($p = 0.09$), and HD and ASSD values were also higher for FSL ($p = 0.85$ and $p = 0.07$, respectively).

Figures S5a and b show the a) 5 female and b) 5 male subjects' ventricular displacement fields for FSL and ANTs as well as the difference between both fields, respectively. Figure S5c shows the averaged displacement field across all 10 randomly selected subjects based on FSL and ANTs as well as the difference plot between both fields. From qualitative comparison at the individual subject level it can be seen that there is remarkable spatial agreement of displacement vector orientation and localization of pronounced displacement magnitudes. There are no blatant differences that would suggest that ANTs is superior to FSL. The difference plot for the overall averaged fields does not show randomly distributed differences across the ventricle but a localization in the anterior section of the ventricle' main body. That being said, we note that the FSL-based registration appears to provide an on average higher ventricular displacement magnitude compared to ANTs with a maximum difference in displacement magnitude of 0.75 mm; however, the majority of the ventricular surface shows minor differences, i.e., mean difference is 0.20 ± 0.15 mm.

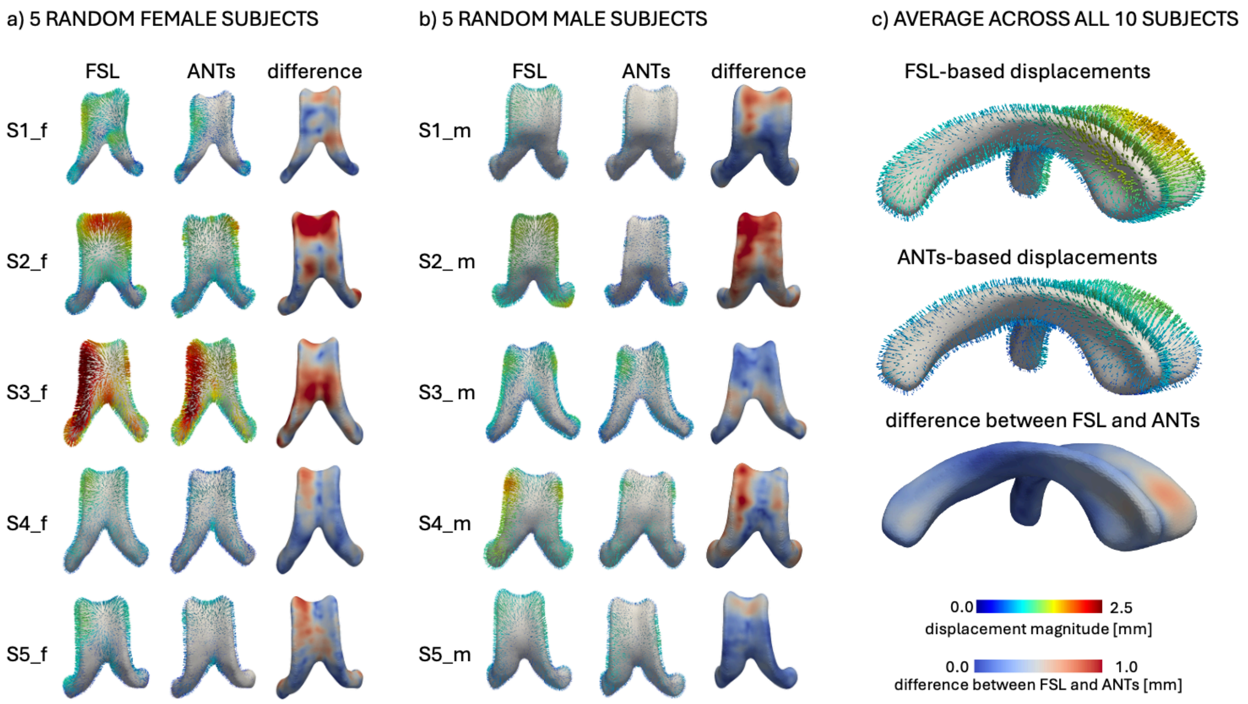

Figure S5: Comparison of registration-derived ventricular displacement fields for a) 5 randomly selected female subjects and b) 5 randomly selected male subjects based on FSL and ANTs as well as the displacement magnitude difference plots, respectively. c) shows the averaged displacement fields for FSL-based and ANTs-based registration results as well as the displacement magnitude difference plot. There are no blatant differences between both codes although we observe that FSL provides a slightly higher overall displacement magnitude compared to ANTs with a mean difference of 0.20 ± 0.15 mm across the whole ventricle.

3. Assessment of hemispheric differences of our mechanomarkers

In order to assess symmetry of the displacement field with respect to left and right hemisphere, we split our model into these two groups based on the ventricle's center of mass. Next, we mirror all nodes of the right hemisphere with respect to the sagittal plane such that the left and right surface mesh overlap. For each node on the left hemisphere we then identify the nearest node on the mirrored right hemisphere to determine a unique list of node-pairs between both hemispheres. The top row of Fig. S6 shows the relative difference between each node-pair for our four mechanomarker fields. We observe that maximum principal wall strain differs most between the two hemispheres while displacement, curvature, and area stretch fields differ less. Specifically, we observe an averaged absolute relative difference of 3.22 ± 4.73 % for maximum principal wall strain, 0.74 ± 1.69 % for curvature, 1.60 ± 2.34 % for displacement, and 0.65 ± 0.95 % for area stretch, respectively. In a second analysis step, we check for node-wise statistically significant difference between left and right hemisphere across our cohort. At each node and for each mechanomarker, we perform a t-test across the cohort and report p-values to indicate locations where left and right hemisphere are statistically significantly different. The bottom row of Fig. S6 shows that the displacement field has the fewest regions with significant differences between hemispheres, i.e., 26% of the ventricular wall. All other markers show more extended wall sections with exhibit hemispheric differences, with ventricular wall fractions of 54% for curvature, 64% for area stretch, and 67% for max principal wall strain, respectively.

Overall, our analysis shows that the ventricular displacement field and area stretch are highly symmetric with respect to hemispheres. Increased differences in the curvature and maximum principal wall strain fields are to be expected as both measures are related to spatial derivatives and, therefore, subject to mesh size and smoothness of the ventricular surface.

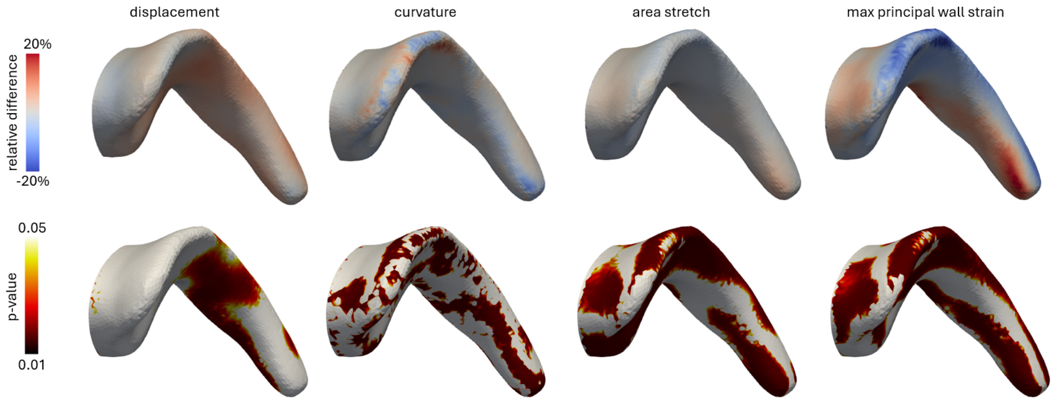

Figure S6: Assessment of hemispheric symmetry. Top row: Relative difference between left and right hemisphere for each of the four mechanomarkers. Displacement magnitude and area stretch show the most similarity between hemispheres while curvature and maximum principal wall strain show more pronounced, albeit spatially-confined differences. Bottom row: p-values from node-wise t-tests establishing cohort-wide significant differences between the left and right hemisphere for each mechanomarker.

4. Inter-subject variability of our mechanomarkers

Figure S7a shows the node-wise variance of the four normalized fields across our 50 subjects. Normalizing each field prior to computing variance enables direct comparison; this shows that nodal displacement magnitude and maximum principal wall strain vary most across our cohort while nodal curvature varies least. Within each marker, largest variances localize along the edges of the ventricle main body as well as the atrium. Figure S7b indicates that the marker's range of variances differs strongly. Specifically, curvature and area stretch are much more homogeneously distributed across the cohort in comparison to displacement and maximum principal wall strain.

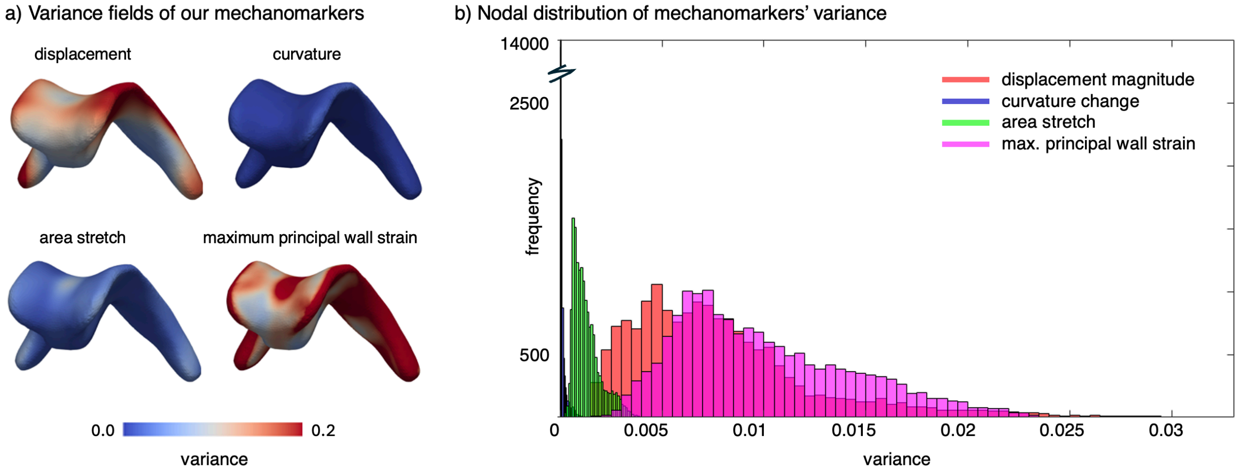

Figure S7: a) Variance of normalized mechanomarkers across the cohort for displacement, curvature, area stretch, and maximum principal wall strain, respectively. b) Histogram of variances of the four markers to provide indication of their homogeneity across the cohort.

To quantify differences between variance fields, we computed the Kullback–Leibler divergence, D_{KL}, which is a measure of how much two different probability distributions are different from each other. More specifically, at each node, we fitted a normal distribution to the variance of each field and determine D_{KL} as follows

$$D_{\mathrm{KL}}(P\|Q) = \sum_{x \in \mathcal{X}} P(x) \log\left(\frac{P(x)}{Q(x)}\right), \tag{1}$$

where P and Q are the probability distributions for the two fields that are being compared. Since the Kullback-Leibler divergence metric is not symmetric, i.e., dependent on the order of probability distributions P and Q, we compute all pairings and show the respective fields in Fig. S8. Larger values of D_{KL} indicate more pronounced differences between the two distributions of variances. As such, dark red regions are associated with more similar variance distributions in comparison to bright sections. Table S1 summarizes the mean with standard deviation of each field comparison. We highlighted the highest values which are consistently associated with the curvature field. These results support our claim that curvature is the most homogeneous field associated with a narrow distribution of variances compared to the other fields.

	displacement	curvature	area stretch	MPWS
displacement	-	**6.70 ± 4.12**	3.00 ± 1.71	2.91 ± 1.61
curvature	**7.37 ± 4.2**	-	**6.81 ± 4.15**	**7.03 ± 3.85**
area stretch	3.39 ± 1.65	**6.09 ± 3.77**	-	2.91 ± 1.72
MPWS	3.49 ± 1.66	**6.49 ± 3.72**	3.04 ± 1.66	-

Table S1: Kullback-Leibler divergence values, D_{KL}, indicating similarities in the variance fields between mechanomarkers. The table is constructed from all P-Q pairings associated with the Kullback-Leibler divergence function (see Eq. 1). Highest numbers are highlighted and consistently associated with the curvature field.

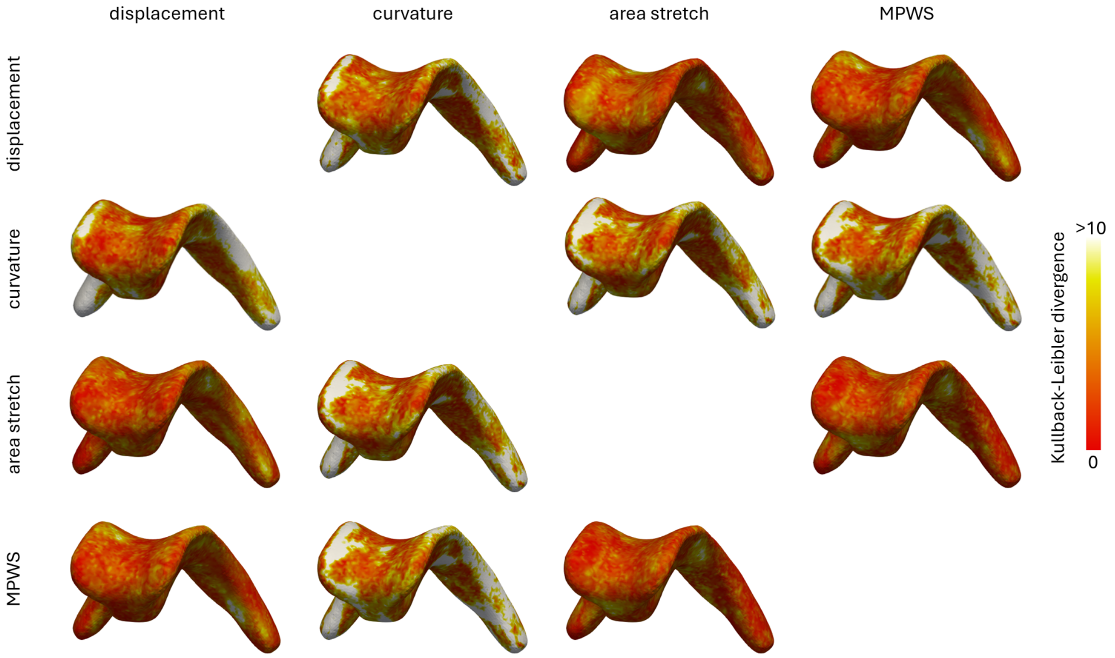

Figure S8: Pairwise Kullback–Leibler divergence, D_{KL}, maps comparing the variance distributions between two mechanomarkers. Since this measure is not symmetric, we compute all permutations for sake of completeness. Larger values, i.e., brighter regions, indicate greater differences in variance distributions between the two mechanomarkers, whereas dark red regions correspond to more similar distributions. These fields indicate that curvature is the most uniform field compared to the other mechanomarkers.
